# Sequentially Activated Smart DNA Nanospheres for Photoimmunotherapy and Immune Checkpoint Blockade

**DOI:** 10.1002/advs.202410632

**Published:** 2024-11-26

**Authors:** Yu Chen, Yu Guo, Wen Cheng, Jiahao Fan, Jiacheng Li, Jiajia Song, Xiaohai Yang, Kemin Wang, Jin Huang

**Affiliations:** ^1^ State Key Laboratory of Chemo/Biosensing and Chemometrics College of Chemistry and Chemical Engineering Key Laboratory for Bio‐Nanotechnology and Molecular Engineering of Hunan Province Hunan University Changsha 410082 P. R. China

**Keywords:** DNA nanospheres, drug delivery, immune checkpoint blockade, photoimmunotherapy, tumor therapy

## Abstract

Due to the inherent immunosuppression and immune evasion of cancer cells, combining photoimmunotherapy with immune checkpoint blockade leverages phototherapy and immune enhancement, overcoming mutual limitations and demonstrating significant anticancer potential. The main challenges include nonspecific accumulation of agents, uncontrolled activation, and drug carrier safety. Smart DNA nanospheres (NS) is developed with targeted delivery and controllable release of photosensitizers and immune agents to achieve effective synergistic therapy and minimize side effects. The multifunctional NS incorporate a targeting module for programming aptamers, a response module for programming i‐motif and DNA/RNA hybrid sequences, and a therapeutic module for packaging photosensitizers and PD‐L1 siRNA. NS navigate to the tumor site and are sequentially activated by intracellular acid and enzymes to release photosensitizers and programmed death ligand 1 (PD‐L1) small interfering RNA (siRNA)^a^. Besides tumor killing and immune promotion, activated NS downregulate PD‐L1 expression, alleviating immune tolerance and evasion, thus enhancing the immune response. These results indicate that NS significantly enhance antitumor immune responses, synergistically improve antitumor efficacy, and reduce systemic toxicity. This study broadens the application of DNA nanomaterials in precision drug delivery and tumor therapy.

## Graphical abstract

Sequentially activated smart DNA nanospheres are developed that incorporate targeting, response, and therapeutic modules to achieve targeted delivery and controlled release of photosensitizers and immune adjuvants. Besides tumor killing, the DNA nanospheres can downregulate PD‐L1 expression, alleviate immune tolerance and escape, enhance immune responses, thereby synergistically improve antitumor efficacy, and reduce systemic toxicity.

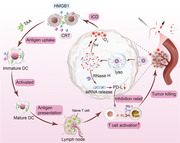

## 1 Introduction

Photoimmunotherapy (PIT) is an innovative cancer treatment that combines phototherapy and immunotherapy. It kills tumor cells through phototherapy while inducing immunogenic cell death (ICD) and anti‐tumor immunity.^[^ ^1^ ^]^ Various recent approaches have explored novel strategies to amplify the immune response and enhance ICD, providing promising insights into improving the efficacy of cancer immunotherapy.^[^ ^2^ ^]^ Compared with chemotherapy or radiotherapy, phototherapy has the advantages of high selectivity, low side effects, localized treatment, and the induction of ICD to enhance immune response. However, due to the inherent immune resistance or immune escape ability of tumor cells,^[^ ^3^ ^]^ it is challenging to completely eradicate the tumor, and the risk of distant metastasis increases.^[^ ^1^, ^4^ ^]^ Therefore, relying solely on photoimmunotherapy may not achieve the best therapeutic effect. The proposed strategy is to combine immune checkpoint blockade (ICB) therapy with PIT, offering potential therapeutic advantages.^[^ ^5^ ^]^ This combination therapy can enhance the immune response, improve immune cell infiltration, overcome immune tolerance, and reduce toxic side effects, thereby enhancing cancer treatment efficacy.

ICB therapy targeting the programmed death protein 1 (PD‐1)/programmed death ligand 1 (PD‐L1) pathway has achieved great success in clinical practice for various tumors.^[^ ^6^ ^]^ Traditional antibody therapies block checkpoints on the cell membrane surface, but the continued expression of intracellular proteins may compensate for immune checkpoints, leading to blockade failure and drug resistance.^[^ ^7^ ^]^ It has been reported that >50% of patients with PD‐L1‐positive tumors experience drug failure.^[^ ^8^ ^]^ PD‐1/PD‐L1 downregulation using small interfering RNA (siRNA) therapy can downregulate expression throughout the entire cell^[^ ^9^ ^]^ and decrease the development of tolerance.^[^ ^10^ ^]^ Therefore, nanocarriers have been designed to simultaneously deliver PD‐L1 siRNA and photosensitizers into tumor cells, combining PIT with ICB to prevent tumor cell resistance and escape, thereby enhancing the therapeutic effect.^[^ ^4^, ^5^, ^11^ ^]^ However, this approach also faces challenges, as the nonspecific accumulation of photosensitizers and immunomodulators after systemic administration may reduce overall efficacy and induce adverse reactions.^[^ ^12^ ^]^ Additionally, uncontrolled activation of photosensitizers and ICB therapeutics can cause systemic phototoxicity and immune‐related adverse events. Although various nanocarriers have been used to co‐package photosensitizers and siRNA, the biocompatibility and biodegradability of drug delivery vehicles may not fully meet the stringent requirements of cancer treatment.^[^ ^13^ ^]^ Therefore, developing novel drug delivery systems to address these issues for synergistic PIT and ICB is highly desirable.

DNA molecules are considered ideal materials for building multifunctional intelligent drug nanocarriers, due to their advantages of programmability, biodegradability, and biocompatibility.^[^ ^14^ ^]^ DNA nanomaterials have become versatile and promising vehicles for drug delivery, significantly promoting the development of precision medicine. Recently, we developed self‐assembled DNA nanospheres (NS) as smart delivery vehicles for precise bioimaging and cancer treatment, due to their high biostability, cell permeability, luge loading capacity, and programmable self–assembly and disassembly behaviors.^[^ ^15^ ^]^ Here, we design a smart self‐assembled NS that can sequentially activate photosensitizers and release PD‐L1 siRNA for efficient synergistic therapy of tumors while reducing side effects on normal tissues. The activated photosensitizers kill tumor cells while inducing ICD to initiate the immune response. The released siRNA downregulates PD‐L1 expression in cancer cells, reducing immune tolerance and escape, thereby enhancing the efficacy of immunotherapy. In this design, the targeting module can be programmed with aptamers to minimize off‐target effects and side effects. The response module can be programmed with i‐motif sequences and DNA/RNA hybrid sequences to develop microenvironment‐activated DNA nanocarriers for controlled activation. The therapeutic module can be packaged with photosensitizers and PD‐L1 siRNA. Briefly, the synergy of phototherapy and immunotherapy can achieve strong tumor inhibition, while the targeted and controllable activation of NS can reduce toxic side effects on normal tissues.

## 2 Results and Discussion

### 2.1 Working Principle

**Scheme 1:**
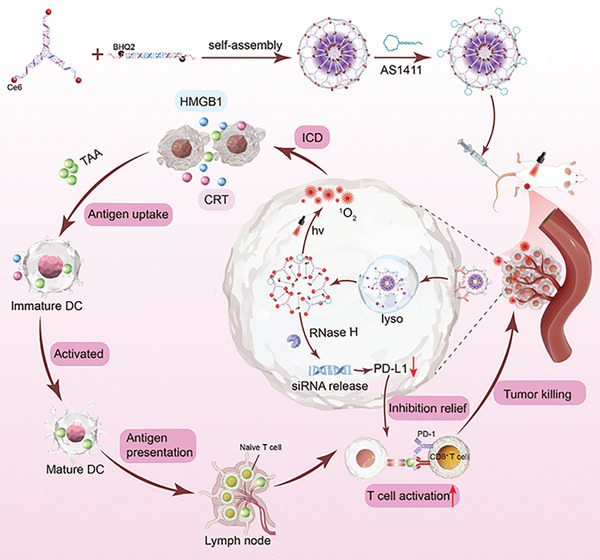
Schematic diagram illustrating the assembly process of NS‐Ce6‐PDL1 and its synergistic effects in cancer photoimmunotherapy and immune checkpoint blockade.

As shown in **Scheme** **1**, the NS was self‐assembled by stepwise hybridization of sticky ends between Y‐shaped DNA monomers (Y) and linker DNA monomers (L). The i‐motif sequence and PD‐L1 siRNA were rationally programmed into the L, and the photosensitizer (chlorin e6, Ce6) and fluorescence quencher (BHQ2) were coupled to the appropriate sites of Y and L, respectively. When Y and L self‐assembled, the photosensitizers were quenched due to their proximity to the quenchers. To enhance the targeting ability, a broad‐spectrum tumor‐specific DNA aptamer (AS1411) was anchored on NS via DNA hybridization. After intravenous injection, the NS was specifically taken up by cancer cells through DNA aptamer targeting. In response to the acidic environment in lysosomes, which led to the conformational change of the i‐motif, the NS would disassemble and escape from lysosomes. This leads to the spatial separation of the photosensitizer and the quencher, producing single‐linear oxygen (^1^O_2_) under laser irradiation, which not only kills tumor cells but also induces cancer cell ICD, thereby activating the immune response. ICD releases signaling molecules such as high mobility group box 1 (HMGB1) and calreticulin (CRT), recruits dendritic cells (DCs) to present tumor‐associated antigens (TAAs), and activates T cells, thereby initiating an immune attack. Meanwhile, RNase H‐mediated siRNA release can downregulate PD‐L1 expression in cancer cells, reducing immune tolerance and escape, thereby enhancing the effectiveness of immunotherapy. Therefore, the synergy of phototherapy and immunotherapy can efficiently kill cancer cells, while the targeted and controllable activation of the NS can minimize toxic side effects on normal tissues.

### 2.2 Self‐Assembly and Disassembly of NS

**Figure 1:**
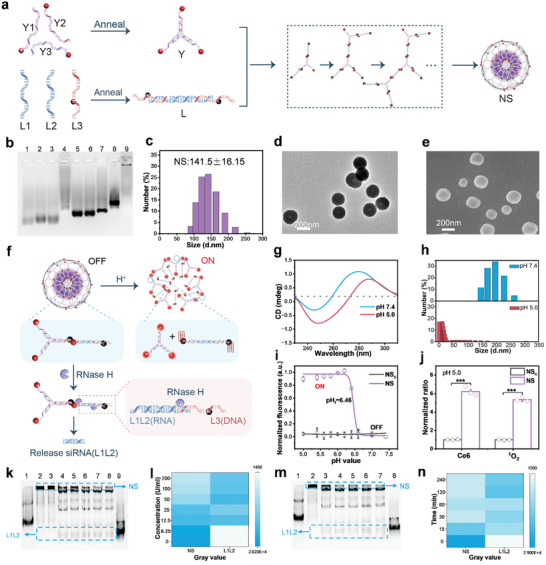
Self‐assembly and disassembly of NS. a) Schematic diagram depicting the self‐assembly of NS. b) 2% agarose electrophoresis analysis of self‐assembly of NS. 1: Y1, 2: Y2, 3: Y3, 4: Y, 5: L1, 6: L2,7: L3, 8: L, 9: NS. c) DLS analysis of NS at pH 7.4 PBS. d) TEM image of NS. e) SEM image of NS. f) Schematic illustration of pH‐mediated and RNase H‐mediated NS disassembly. g) CD spectra of NS at pH 7.4 and pH 5.0. h) Particle size analysis of NS at pH 7.4 and 5.0 by DLS. i) The acid response intervals of NS_c_ and NS. j) Ratio of Ce6 fluorescence recovery and ^1^O_2_ generation in NS_c_ and NS at pH 5.0. k) 10% PAGE analysis of NS‐Ce6‐PDL1 after incubation with different concentrations of RNase H at 37 °C. 1: Y, 2: NS, 3–8: NS + RNase H(U mL^−1^), 3: 6.25, 4: 12.5, 5: 25, 6: 50, 7: 100, 8: 200, 9: L1L2. l) Image‐J analyzes the gray values of the NS and L1L2 bands in Figure (k). m) 10% PAGE analysis of NS‐Ce6‐PDL1 after incubation with 100U mL^−1^ RNase H for various durations at 37 °C. 1: Y, 2: NS, 3: 15 min, 4: 30 min, 5: 60 min, 6: 120 min, 7: 240 min, 8: L1L2. n) Image‐J analysis of gray values of NS and L1L2 bands in Figure (m). Results are presented as means ± SD (*n* = 3) (***p < 0.001; calculated by t‐test).

NS was synthesized through a two‐step self‐assembly process (**Figure** **1**a): i) assembly of Y and L DNA monomers, and ii) hybridization of the sticky ends between Y and L to form NS (Figure S1 and Table S1, Supporting Information). Specifically, three oligonucleotides (Y1, Y2, and Y3) were annealed to synthesize the Y monomer, with each end modified with Ce6. Similarly, three oligonucleotides (L1, L2, and L3) were annealed to synthesize the L monomer, with PD‐L1 siRNA incorporated into the L1L2 duplex, and the i‐motif sequence and BHQ2 incorporated into L3. Subsequently, the Y monomer, containing three sticky ends, hybridized with the L monomer, containing two complementary sticky ends, forming branched DNA and ultimately synthesizing NS. Agarose gel electrophoresis analysis confirmed the stepwise self‐assembly of NS (Figure 1b). The band in lane 4 indicated the assembly of Y from Y1, Y2, and Y3 (lanes 1–3). Similarly, the band in lane 8 indicated the assembly of L from L1, L2, and L3 (lanes 5–7). The bands in lane 9 demonstrated the successful stepwise self‐assembly of NS from Y and L monomers. Electrophoretic fluorescence images showed that the fluorescence of Ce6 was effectively quenched by BHQ2 (Figure S2, Supporting Information). Optimization experiments indicated that the incubation time for synthesizing NS was over 24 h (Figure S3, Supporting Information) and the Y/L ratio was 1:2 (Figure S4, Supporting Information). Characterization of the optimized NS revealed an average particle size of ≈141.5 nm, as measured by dynamic light scattering (DLS) (Figure 1c). The NS were spherical structures and relatively uniform, as analyzed by transmission electron microscopy (TEM) (Figure 1d) and scanning electron microscopy (SEM) (Figure 1e). To verify the stability of NS, they were incubated in phosphate‐buffered saline (PBS) and 10% fetal bovine serum (FBS) for various periods. Agarose gel electrophoresis results showed no significant degradation of NS and L1L2 duplex in PBS (Figure S5, Supporting Information). However, degradation bands for L1L2 were observed after 0.5 day of incubation in 10% FBS, whereas NS bands remained visible after 4 days, suggesting that incorporating siRNA (L1L2) into NS provides some protection to the RNA (Figure S6, Supporting Information).

The acid‐mediated and RNase H‐mediated disassembly of NS is illustrated in Figure 1f. In detail, acidic conditions can induce C‐rich sequences to form i‐motif structures. This transformation disrupts the hybridization between Y and L, resulting in the disassembly of NS (Figure S7, Supporting Information). Circular dichroism (CD) spectroscopy results indicated that the characteristic peaks of i‐motif were red‐shifted due to the formation of the i‐motif structure at pH 5.0 compared with pH 7.4 (Figure 1g). The DLS results showed a decrease in the average size of NS at pH 5.0 compared to pH 7.4 (Figure 1h). In addition, we verified the ¹O₂ generation of NS under acidic conditions by electron spin resonance (ESR) (Figure S8, Supporting Information). The results showed that compared with pH 7.4, the production of ¹O₂ in NS increased significantly at pH 5.0, and its production gradually increased with the extension of illumination time. To further investigate the acid‐activated disassembly of NS, we prepared non‐acid‐responsive control NS (NS_c_) by changing the C base to T base and incubating NS and NS_c_ with PBS of different pH. The results showed that with the increase of acid, the fluorescence of NS changed from “OFF” to “ON”, and NS_c_ always remained “OFF” (Figure 1i). In addition, the results showed that the pH transition midpoint (pH_t_) value of NS was 6.46 (Figure 1i). It suggested that NS could disassemble and activate the photosensitizer during the maturation phase of the cell lysosome (pH 4.5–6.8). Kinetic analysis showed that the fluorescence of NS was recovered rapidly within ≈1.5 h at pH 5.0, whereas the fluorescence of NS_c_ was barely recovered within 6 h (Figure S9, Supporting Information). With the disassembly of NS, Ce6, and BHQ2 were separate from each other, leading to fluorescence recovery and ^1^O_2_ generation. Fluorescence analysis of Ce6 and single‐linear oxygen sensor green (SOSG) probe revealed that under acidic conditions, Ce6 fluorescence recovery and ^1^O_2_ generation were significantly higher in NS compared to NS_c_ (Figure 1j).

In addition to the generation of ^1^O_2_ by acid‐mediated NS disassembly, RNase H‐mediated siRNA release is a crucial step in ICB. RNase H is known to recognize DNA/RNA hybrid duplexes and selectively degrade the RNA fragments.^[^ ^16^ ^]^ NS assembly relies on the sticky end hybridization between the Y monomer and the siRNA linker monomer (L), forming a DNA/RNA duplex. In the presence of RNase H, the RNA fragments of the DNA/RNA duplex could be degraded, leading to the release of siRNA duplex (Figure 1f). As shown in Figure 1k and Figure S10 (Supporting Information), after incubating NS with different concentrations of RNase H, the degradation of NS and the release of PD‐L1 siRNA (L1L2) could be observed, which would downregulate the target mRNA in tumor cells. The gray value quantification of NS and L1L2 bands indicated that L1L2 duplex could be effectively released from NS at an RNase H concentration of 50 U mL^−1^ (Figure 1l). Additionally, it was found that the efficiency of RNase H‐mediated NS disassembly was time‐dependent (Figure 1m; Figure S10, Supporting Information). Quantitative results showed that L1L2 duplex could be effectively released from NS in ≈30 min (Figure 1n). The above results indicated that NS could be directly disassembled to release siRNA in the presence of RNase H.

### 2.3 Cellular Uptake and Lysosomal Escape

**Figure 2:**
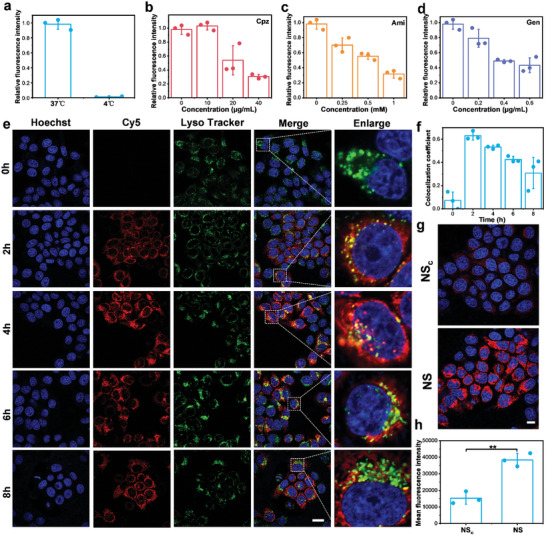
Cellular uptake behavior of NS. a) Quantification of the fluorescence of Cy5‐NS uptake by MCF‐7 cells under different temperatures. b–d) Quantification of the fluorescence of Cy5‐NS uptake by MCF‐7 cells after treatment with different concentrations of endocytosis inhibitors: b) Chlorpromazine (CPZ), c) Amiloride (Ami), d) Genistein (Gen). e) CLSM images of MCF‐7 cells after incubation with Cy5‐NS for varying durations. Cell nuclei stained with Hoechst 33342 (blue) and lysosomes stained with LysoTracker (green). Scale bar = 20 µm. f) Quantitative analysis of co‐localization coefficients in (e). g) CLSM images of MCF‐7 cells incubated with Cy5‐NS and Cy5‐NS_c_ for 8 h, respectively. Scale bar = 10 µm. h) Quantitative analysis of the relative fluorescence intensity in (g). Results are presented as means ± SD (*n* = 3) (**p < 0.01; calculated by t‐test).

Next, we investigated the cellular uptake of NS in MCF‐7 cells. To enhance the tumor‐targeting capacity, DNA aptamer AS1411 with a complementary tail was anchored on NS. First, cellular uptake studies of NS were performed using confocal laser scanning microscopy (CLSM) and flow cytometry. Two types of Cy5‐labeled NS with and without aptamer (NS (+apt) and NS (−apt)) were incubated with MCF‐7 cells and then analyzed by CLSM (Figure S11, Supporting Information) or flow cytometry (Figure S12, Supporting Information) at various time points. The results consistently showed that NS (+apt) was more rapidly taken up by cells than NS (−apt). In addition, we found that the cellular uptake of NS was concentration‐dependent (Figure S13, Supporting Information). Second, the cellular uptake behavior of NS was investigated through a series of cell biology experiments. At low temperatures, active energy‐dependent endocytosis processes were inhibited.^[^ ^17^ ^]^ Therefore, we incubated Cy5‐NS with MCF‐7 cells at 4 and 37 °C, respectively, and subsequently imaged the fluorescence of Cy5 using CLSM. The results showed that the fluorescence of Cy5‐NS decreased to almost zero at 4 °C compared to 37 °C (**Figure** **2**a; Figure S14, Supporting Information). To investigate the endocytosis pathway of NS, MCF‐7 cells were pretreated with varying concentrations of specific endocytosis inhibitors. The results indicated that chlorpromazine (Cpz, clathrin‐mediated endocytosis inhibitor), amiloride (Ami, macropinocytosis inhibitor), and genistein (Gen, caveolae‐mediated endocytosis inhibitor) all inhibited endocytosis in a dose‐dependent manner (Figure 2b–d; Figure S15, Supporting Information). These results suggested that cellular uptake of NS was an energy‐dependent process and relies on micropinocytosis, clathrin‐mediated endocytosis, and caveolae‐mediated endocytosis.^[^ ^18^ ^]^ The endocytosis pathway indicated that NS could be translocated into the cytoplasm via the lysosomal pathway.

To investigate whether NS could escape from lysosomes to avoid enzymatic degradation, we used CLSM to track the co‐localization between Cy5‐labeled NS and lysosomes (Figure 2e). After the NS entered cells, the fluorescence signals of Cy5 and LysoTracker significantly overlapped at 2 h, suggesting that NS had been translocated to lysosomes. After 4 h of incubation, the fluorescence intensity of intracellular Cy5 increased and the fluorescence co‐localization coefficient with LysoTracker decreased (Figure 2f), indicating that NS could escape into the cytoplasm. After 6 h, the co‐localization coefficient of NS with lysosomes further decreased (Figure 2f). In the merge image, we can see that the lysosomal green fluorescence at 6 and 8 h exists independently (Figure 2e), suggesting that most of the NS had transferred to the cytoplasm after escaping from the lysosome. To verify the degree of activation of NS and NS_c_ within cells, we used CLSM to image the fluorescence of Cy5 after incubating with MCF‐7 cells for the same duration. The results showed that the activation extent of NS was significantly higher than that of NS_c_ (Figure 2g,h). These results suggested that the acidic environment in the lysosome could activate NS and facilitate its escape, thereby reducing the degradation of siRNA by the lysosome.

### 2.4 In Vitro Cancer Cell Killing Effects

**Figure 3:**
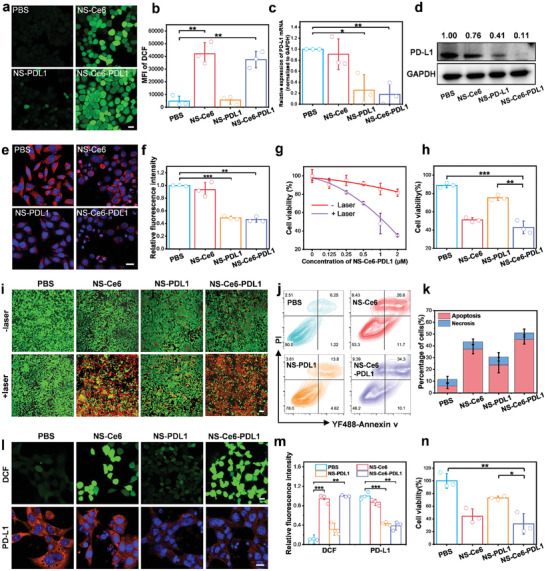
In vitro synergistic cancer cell killing effects. a) Detection of ^1^O_2_ in MCF‐7 cells using DCFH‐DA probe after different treatments. Scale bar = 20 µm. b) Quantification of mean fluorescence intensity (MFI) in (a). c) RT‐qPCR analysis of PD‐L1 mRNA expression in MCF‐7 cells after different treatments. d) Western blot analysis of PD‐L1 protein expression in MCF‐7 cells after different treatments. e) IF analysis of PD‐L1 expression in MCF‐7 cells after different treatments. Scale bar = 20 µm. f) Quantitative analysis of relative fluorescence intensity in (e). g) MTS analysis of cell viability of MCF‐7 cells treated with different concentrations of NS‐Ce6‐PDL1 with or without laser irradiation. h) MTS analysis of cell viability of MCF‐7 cells after different treatments. i) Calcein/PI staining of MCF‐7 cells after different treatments with or without laser irradiation. Living and dead cells stained with calcein‐AM (green) and PI (red), respectively. Scale bar = 100 µm. j) Flow cytometry analysis of YF488‐Annexin v/PI staining in MCF‐7 cells after different treatments. k) Quantitative analysis of apoptosis and necrosis in (j). l) ^1^O_2_ generation and PD‐L1 expression in 4T1 cells after different treatments. Scale bar = 20 µm. m) Quantitative analysis of the relative fluorescence intensity in (l). n) MTS analysis of cell viability of 4T1 cells after different treatments. Results are presented as means ± SD (*n* = 3) (*p < 0.05, **p < 0.01, ***p < 0.001; calculated by t‐test).

To further evaluate the ^1^O_2_‐inducing effect of NS on MCF‐7 cells under laser irradiation, we used DCFH‐DA as an intracellular ^1^O_2_ detection probe. It could generate fluorescent DCF within cells for ^1^O_2_ detection.^[^ ^19^ ^]^ CLSM results showed that NS‐Ce6 and NS‐Ce6‐PDL1 groups produce significant green fluorescence after laser irradiation in cells (**Figure** **3**a,b). To investigate whether siRNA could be released from NS and reduce PD‐L1 expression, we first evaluated the silencing effect of siPD‐L1 by immunofluorescence (IF) and RT‐qPCR, using scrambled siRNA as a negative control (NC). The results showed that siRNA against PD‐L1 (siPD‐L1) significantly reduced PD‐L1 expression in MCF‐7 cells compared to the PBS and NC groups (Figure S16, Supporting Information). Next, the expression of PD‐L1 was evaluated using RT‐qPCR and Western blot (WB) after incubating MCF‐7 cells with different NS formulations. The results showed a significant reduction in PD‐L1 mRNA levels (Figure 3c) and protein levels (Figure 3d) in the NS‐PDL1 and NS‐Ce6‐PDL1 groups compared to the PBS group. Further IF analysis demonstrated a significant decrease in PD‐L1 expression in cells treated with NS‐PDL1 and NS‐Ce6‐PDL1 (Figure 3e,f). These results indicate that NS was activated in cells, produced ^1^O_2_ and downregulated PD‐L1 expression.

Meanwhile, the results of the phototoxicity test indicated that cell survival was largely unaffected by 100 mW cm^−^^2^ laser irradiation for up to 10 min (Figure S17, Supporting Information). Next, cytotoxicity assays were conducted to investigate the phototoxicity of NS‐Ce6‐PDL1 with or without irradiation. In the absence of irradiation, NS‐Ce6‐PDL1 exhibited no significant cytotoxic effect on MCF‐7 cells. However, under laser irradiation, NS‐Ce6‐PDL1 significantly induced cell death in a dose‐dependent manner (Figure 3g), indicating its notable photodynamic efficacy. After treating MCF‐7 cells with different NS formulations, cell viability results indicated that the NS‐Ce6‐PDL1 group exhibited the strongest cancer cell killing effect (Figure 3h). Additionally, the results of calcein/PI staining intuitively showed that both NS‐Ce6 and NS‐Ce6‐PDL1 groups had strong cancer cell killing ability (Figure 3i). Flow cytometric analysis of YF488‐Annexin V/PI staining showed that the percentages of apoptosis and necrosis in cells treated with NS‐Ce6 and NS‐Ce6‐PDL1 were significantly higher than those in the control group (Figure 3j,k). NS‐PDL1‐treated cells exhibited a higher proportion of apoptotic cells, possibly due to the silencing of PD‐L1, which may induce downstream STAT3 phosphorylation and subsequently trigger apoptosis.^[^ ^20^ ^]^ Furthermore, we investigated the internalization of NS‐Ce6‐PDL1 and its cytotoxic effects on normal cells (MCF‐10A). The results showed that the uptake of NS by MCF‐10A cells was significantly reduced compared to MCF‐7 cells, and the cytotoxic effect of NS‐Ce6‐PDL1 on MCF‐10A cells was lower than on MCF‐7 cells (Figures S18 and S19, Supporting Information). It suggested that the inefficient uptake of NS‐Ce6‐PDL1 had lower cytotoxic effects on normal tissues.

To investigate the generality of NS‐Ce6‐PDL1 in silencing PD‐L1 and inducing phototoxic cancer cell killing, we conducted similar experiments using mouse‐derived breast cancer cells (4T1). First, we investigated the cellular uptake of NS (+apt) and NS (−apt) in 4T1 cells using CLSM and flow cytometry. The results indicated that NS (+apt) was taken up by the cells more rapidly compared to NS (−apt) (Figures S20 and S21, Supporting Information). Second, we used CLSM to detect ^1^O_2_ generation and PD‐L1 expression in 4T1 cells. The CLSM images analyzed with the DCFH‐DA probe showed significantly higher ^1^O_2_ levels in the NS‐Ce6 and NS‐Ce6‐PDL1 groups, suggesting that the activation of the photosensitizer further induced ^1^O_2_ production (Figure 3l,m). CLSM images from IF analysis showed that PD‐L1 expression was significantly suppressed in the NS‐PDL1 and NS‐Ce6‐PDL1 groups, indicating that the release of siPD‐L1 further downregulated PD‐L1 protein expression (Figure 3l,m). After treating 4T1 cells with different NS formulations, cell viability results indicated that the NS‐Ce6‐PDL1 group exhibited the strongest cancer cell killing effect (Figure 3n). These results indicated that NS‐Ce6‐PDL1 could similarly release PD‐L1 siRNA and generate ^1^O_2_ in 4T1 cancer cells, exerting a potent cancer cell killing effect.

### 2.5 In Vitro Induction of Immunogenic Cell Death

**Figure 4:**
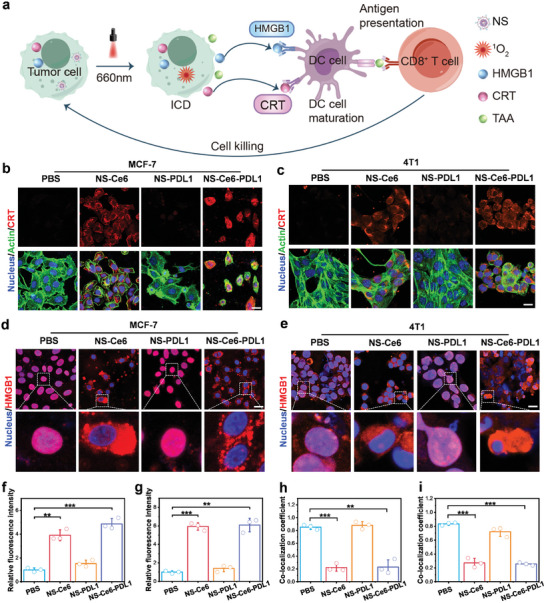
In vitro induction of immunogenic cell death. a) Schematic representation of ICD‐induced T‐cell killing of cancer cells. b) IF staining analysis of CRT release in MCF‐7 cells after different treatments. Cells co‐stained with Nucleus (blue), Actin (green), and CRT (red). Scale bar = 20 µm. c) IF staining analysis of CRT release in 4T1 cells after different treatments. Scale bar = 20 µm. d) IF staining analysis of HMGB1 release in MCF‐7 cells after different treatments. Cells co‐stained with Nucleus (blue) and HMGB1 (red). Scale bar = 20 µm. e) IF staining analysis of HMGB1 release in 4T1 cells after different treatments. Scale bar = 20 µm. f) Quantitative analysis of relative fluorescence intensity in (b). g) Quantitative analysis of relative fluorescence intensity in (c). h) Co‐localization coefficients of HMGB1 and nucleus in (d) quantified using Image J. i) Co‐localization coefficients of HMGB1 and nucleus in (e) quantified using Image J. Results are presented as means ± SD (*n* = 3) (*p < 0.05, **p < 0.01, ***p < 0.001; calculated by t‐test).

NS‐treated tumor cells were exposed to light to generate ^1^O_2_. In addition to directly killing tumor cells, it could further trigger an immune response by inducing immunogenic cell death (**Figure** **4**a). During this process, tumor cells killed by phototherapy could release TAAs and damage‐associated molecular patterns (DAMPs). DAMPs act as “danger” signals that stimulate the immune system, inducing the maturation of DCs.^[^ ^21^ ^]^ It promotes the presentation of TAAs by DCs and the activation of T‐cells, thereby initiating a cytotoxic T‐cell (CD8^+^) attack on the tumor cells. To evaluate the ability of NS‐Ce6‐PDL1 to induce ICD in cancer cells, we determined the release or exposure of key DAMPs, including CRT and HMGB1. CLSM imaging results showed that NS‐Ce6 and NS‐Ce6‐PDL1 treatment caused surface exposure of CRT in MCF‐7 and 4T1 cells (Figure 4b,c). Additionally, after NS‐Ce6 and NS‐Ce6‐PDL1 treatment, the HMGB1 translocated from the nucleus to the cytoplasm or extracellular environment in MCF‐7 and 4T1 cells (Figure 4d,e; Figures S22 and S23, Supporting Information). The relative expression of CRT (Figure 4f,g) and the co‐localization coefficients of nuclei and HMGB1 (Figure 4h,i) in MCF‐7 cells and 4T1 cells were further quantified. These results further confirm the occurrence of ICD mediated by the photodynamic effect.

### 2.6 In Vitro Promotion of T‐Cell Activation and Killing

**Figure 5:**
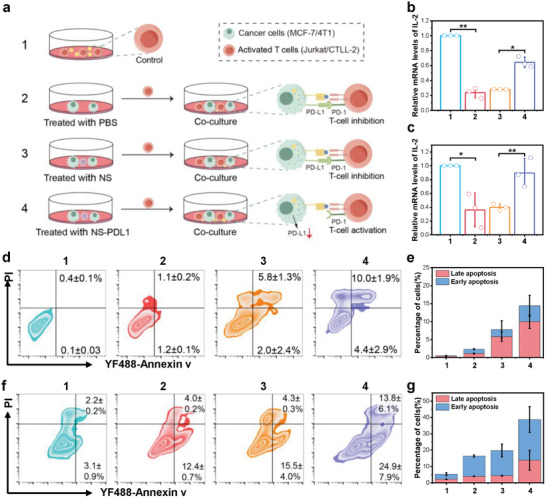
In vitro promotion of T‐cell activation and killing. a) Schematic representation of cancer cells co‐cultured with T‐cells. b) RT‐qPCR analysis of IL‐2 mRNA levels in Jurkat T‐cells in a co‐culture experiment with MCF‐7 cells. c) RT‐qPCR analysis of IL‐2 mRNA levels in CTLL‐2 cells in a co‐culture experiment with 4T1 cells. d) YF488‐Annexin V/PI analysis of apoptosis in MCF‐7 cells in a co‐culture experiment with Jurkat T‐cells. e) Quantitative analysis of late apoptosis and early apoptosis in (d). f) YF488‐Annexin V/PI analysis of apoptosis in 4T1 cells in a co‐culture experiment with CTLL‐2 cells. g) Quantitive statistics of the late apoptosis and early apoptosis in (f). Results are presented as means ± SD (*n* = 3) (*p < 0.05, **p < 0.01; calculated by t‐test).

To investigate whether NS‐mediated siRNA release can silence PD‐L1 expression and thereby promote T‐cell activation and cancer cell killing, we conducted in vitro co‐culture experiments with cancer cells and T‐cells. As shown in **Figure** **5**a, we set up four groups: Group 1 consisted of activated T‐cells alone, which could release a large amount of Interleukin‐2 (IL‐2), and served as a control. Groups 2, 3, and 4 involved cancer cells pretreated with PBS, NS, and NS‐PDL1, respectively, followed by co‐culture with activated T‐cells. These setups resulted in varying degrees of T‐cell activation and IL‐2 release. We measured IL‐2 levels in the two co‐culture models to assess T‐cell activation. First, we co‐cultured MCF‐7 cells with phytohemagglutinin (PHA)‐stimulated Jurkat T‐cells. The upregulation of IL‐2 confirmed PHA‐induced Jurkat T‐cell activation (Figure S24, Supporting Information). Co‐culturing activated Jurkat T‐cells with MCF‐7 cells resulted in decreased IL‐2 mRNA levels, but this reduction could be reversed by treating the tumor cells with NS‐PDL1 (Figure 5b). Second, similar experiments were conducted with 4T1 cells and mouse‐derived cytotoxic T cells (CTLL‐2). We confirmed the activation of CTLL‐2 (Figure S25, Supporting Information). Then, CTLL‐2 was co‐cultured with 4T1 cells, and IL‐2 expression levels in CTLL‐2 cells were measured. Consistent with previous results, IL‐2 levels in CTLL‐2 cells co‐cultured with 4T1 cells decreased, but this reduction could be reversed by NS‐PDL1 treatment of the tumor cells (Figure 5c).

To evaluate the effect of NS‐PDL1 on T‐cell‐mediated tumor cell killing, DiD‐stained MCF‐7 cells were co‐cultured with activated Jurkat T‐cells. CLSM images and statistical analyses showed that the fluorescence intensity of NS‐PDL1‐pretreated MCF‐7 cells significantly decreased compared to the PBS and NS treatment groups (Figure S26, Supporting Information). This result indicated a significant killing effect of Jurkat T‐cells on MCF‐7 cells with low PD‐L1 expression. Furthermore, apoptosis induced by NS in co‐cultured cells was assessed using flow cytometry with YF488‐Annexin V/PI staining (Figure 5d,e). After co‐culturing with Jurkat cells, the percentage of early and late apoptotic cells in the NS‐PDL1 treatment group was ≈15%, higher than in the other groups. Similarly, cell‐killing experiments using 4T1 cells co‐cultured with CTLL‐2 cells demonstrated that CTLL‐2 cells significantly killed 4T1 cells with low PD‐L1 expression (Figure S27, Supporting Information). Consistently, flow cytometry apoptosis assays showed the highest apoptosis rate in 4T1 cells in the NS‐PDL1 treatment group (Figure 5f,g). Notably, CTLL‐2 cells exhibited a better killing effect on 4T1 cells compared to Jurkat cells on MCF‐7 cells, likely because CTLL‐2 cells are cytotoxic T‐cells that can directly kill cancer cells. These results collectively indicated that NS‐PDL1 reversed immunosuppression by downregulating PD‐L1 in cancer cells, thereby promoting T‐cell activation and enhancing T‐cell anti‐tumor activity.

### 2.7 In Vivo Imaging and Synergistic Antitumor Immunity

To investigate the tumor‐targeting ability and in vivo biodistribution of NS, we established a 4T1 tumor xenograft model in BALB/c mice. We studied the time‐dependent in vivo biodistribution of Cy5‐labeled NS with “always‐on” signals (NS (+apt) and NS (−apt)) through tail vein injection (Figure S28, Supporting Information). Imaging and statistical results showed that NS (−apt) had low accumulation efficiency and was rapidly cleared after injection, whereas NS (+apt) reached its highest accumulation efficiency at 0.5 h post‐injection and maintained a high tumor retention rate even after 24 h. This indicated that the multivalent aptamers anchored on the NS surface allow it to specifically target and be rapidly internalized and delayed cleared by 4T1 cells. After 24 h, the mice were dissected, and the biodistribution of NS in major organs (heart, liver, spleen, lung, kidney) and tumor tissue was analyzed (Figure S29, Supporting Information). The results showed that NS could be metabolized by the liver and kidney, and NS (+apt) significantly accumulated in tumor tissue compared to NS (−apt). Additionally, time‐dependent fluorescence images of tumor sections also demonstrated that NS (+apt) had a relatively long tumor retention capability (Figure S30, Supporting Information). These results suggested that NS could specifically accumulate at tumor sites, thereby enhancing drug efficacy under laser irradiation while reducing systemic toxicity. In addition, we also studied the activation imaging of different NS in BALB/c nude mice by in situ injection. The results showed that the fluorescence intensity of NS after injection was always higher than that of NS_c_ (Figure S31, Supporting Information). This indicated that NS achieved good activation imaging at the tumor site.

**Figure 6:**
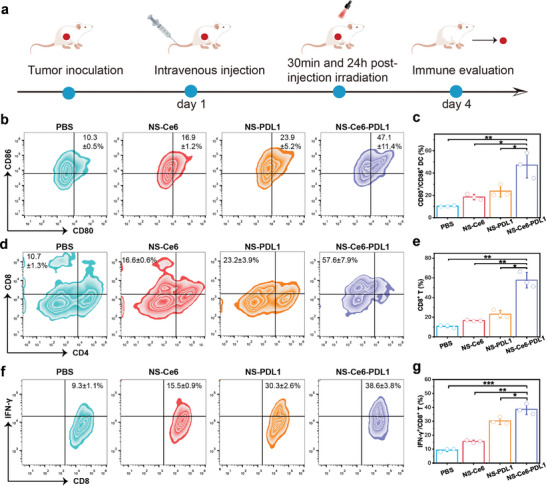
In vivo synergistic antitumor immunity. a) Experimental protocol for evaluating in vivo antitumor immunity. b) Analysis of CD80^+^/CD86^+^ dendritic cells using flow cytometry following various treatments. c) Quantitative analysis of the frequency of CD80^+^/CD86^+^ in (b). d) Analysis of tumor‐infiltrating CD8^+^ T‐cells using flow cytometry following various treatments. e) Quantitative analysis of the frequency of CD8 ^+^ T‐cells in (d). f) Analysis of CD8^+^/IFN‐γ^+^ T‐cells in tumors using flow cytometry following various treatments. g) Quantitative analysis of the frequency of CD8^+^/IFN‐γ^+^ T‐cells in (f). Results are presented as means ± SD (*n* = 3) (*p < 0.05, **p < 0.01, ***p < 0.001; calculated by t‐test).

Next, to evaluate the synergistic efficiency of NS‐Ce6‐PDL1‐based photodynamic immunotherapy, we established a 4T1 tumor‐bearing model and monitored key immune cells by flow cytometry to study the synergistic effects of the immunotherapy. When the xenograft tumors reached ≈300 mm^3^, different NS formulations were injected into the mice via the tail vein, and the experimental process is shown in **Figure** **6**a. Four groups of mice were randomly assigned, and they received treatments of PBS, NS‐Ce6, NS‐PDL1, and NS‐Ce6‐PDL1. According to the mechanisms reported in the literature, ICD‐induced TAAs can be taken up and phagocytosed by immature DCs, leading to DCs maturation.^[^ ^22^ ^]^ Subsequently, mature DCs present TAAs to T‐cells in the lymph nodes, and activate the T‐cells. Therefore, three days after the injection of different formulations, we dissected the inguinal lymph nodes of 4T1 tumor‐bearing mice and collected DCs. After staining the cells with corresponding antibodies(anti‐CD11c/anti‐CD80/anti‐CD86), the frequency of mature DCs was measured by flow cytometry. As shown in Figure 6b,c, compared to the PBS‐treated group (10.3% ± 0.5%), the frequency of mature DCs in NS‐Ce6 and NS‐PDL1 groups were increased to 16.9% ± 1.2% and 23.9% ± 5.2%, respectively, which were 1.64‐ and 2.32‐fold higher compared to the PBS group. In contrast, the NS‐Ce6‐PDL1 group exhibited the highest frequency of DC maturation at 47.1% ± 11.4%, which was 4.57‐fold higher compared to the PBS group. This indicated that the NS‐based PIT/ICB combination therapy significantly promoted DC maturation compared to monotherapies.

After T‐cell activation, cytotoxic T‐cells (CD8^+^) can infiltrate tumor tissues and kill tumor cells by secreting toxic cytokines such as interferon‐γ (IFN‐γ), granzyme, and perforin.^[^ ^23^ ^]^ Therefore, we further extracted tumor‐infiltrating lymphocytes, stained with corresponding antibodies (anti‐CD3/anti‐CD4/anti‐CD8), and measured the infiltrating CD8^+^ cells using flow cytometry (Figure 6d,e). The results showed that PIT and siPD‐L1 treatment alone (NS‐Ce6 and NS‐PDL1 groups) increased the frequency of CD8^+^ cells to 16.6% ± 0.6% and 23.2% ± 3.9%, respectively, which were 1.55‐ and 2.17‐fold higher compared to the PBS group. Furthermore, the combination therapy group (NS‐Ce6 ‐PDL1) exhibited the highest frequency (57.6% ± 7.9%) of CD8^+^ cells in the tumor tissue, which was 5.38‐fold higher compared to the PBS group. We also assessed the expression of IFN‐γ in CD8^+^ T‐cells by staining with anti‐CD3, anti‐CD8, and anti‐IFN‐γ antibodies (Figure 6f,g). Similarly, compared to the PBS group, PIT and siPD‐L1 treatment alone increased the frequency of CD8^+^/IFN‐γ^+^ double‐positive T‐cells to 15.5% ± 0.9% and 30.3% ± 2.6%, respectively. In contrast, the combination therapy group showed a higher frequency of CD8^+^/IFN‐γ^+^ double‐positive T‐cells (38.6% ± 3.8%). These results indicated that, compared to monotherapies, the NS‐based PIT/ICB combination therapy could induce a stronger immune response, suggesting its potential to enhance antitumor efficacy.

### 2.8 In Vivo Synergistic Antitumor Effects

**Figure 7:**
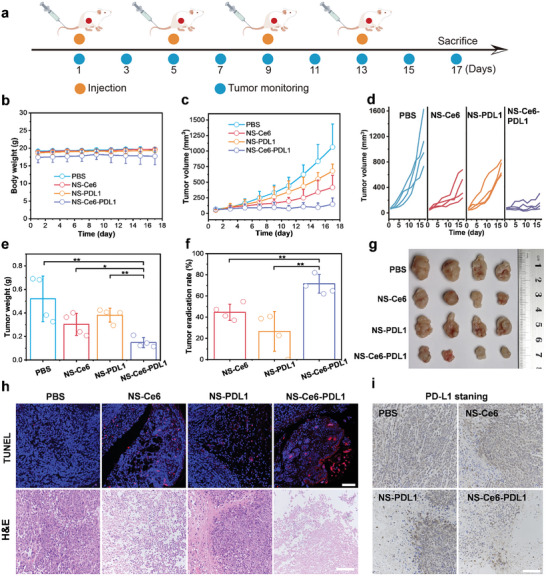
In vivo synergistic antitumor effects. a) Protocol for establishing the 4T1 tumor xenograft model and treatment procedures. b) Body weight of 4T1 tumor‐bearing mice after different treatments. c) Tumor growth curve of 4T1 tumor‐bearing mice after different treatments. d) Individual tumor growth curves of 4T1 tumor‐bearing mice after different treatments. e) Tumor weights of mice at the end of different treatments. (f) Tumor eradication rates relative to the PBS control at the end of different treatments. g) Photograph of the tumor at the end of different treatments. h) TUNEL and H&E staining of tumor at the end of different treatments. Scale bar  = 100 µm. i) Immunohistochemical analysis of PD‐L1 expression in tumors at the end of different treatments. Scale bar  = 100 µm. Results are presented as means ± SD (*n* = 4) (*p < 0.05, **p < 0.01; calculated by t‐test).

The in vivo synergistic tumor treatment effect was then investigated using 4T1 tumor‐bearing mice. Following the protocol shown in **Figure** **7**a, the 4T1 tumor‐bearing mice were randomly divided into four different drug‐treatment groups (PBS, NS‐Ce6, NS‐PDL1, and NS‐Ce6‐PDL1) (*n* = 4). The drug formulations were intravenously injected every two days for a total of four injections, with tumor weight and volume monitored on alternate days. Tumors were irradiated with a 660 nm laser (100 mW cm^−^^2^, 5 min) at 30 min and 24 h after each administration. The results showed no significant changes in the body weight of the mice during treatment, indicating that the different formulations were biologically safe (Figure 7b). The tumor growth curves showed that compared to the PBS group, monotherapies including NS‐Ce6 and NS‐PDL1 treatments could delay tumor growth to some extent. In contrast, NS‐Ce6‐PDL1 treatment exhibited the highest antitumor efficiency, with the tumor growth curve tending to plateau (Figure 7c,d). At the end of treatment, the excised tumors were weighed and photographed. As shown in Figure 7e, the combination therapy group displayed the smallest tumor mass. However, the tumor eradication rates for PIT and siPD‐L1 treatments were 40% and 20%, respectively, while the combination therapy group exhibited a higher tumor eradication rate of 65% (Figure 7f). The difference in tumor sizes visually demonstrated that the combination therapy group had the best tumor suppression effect (Figure 7g). The NS‐Ce6‐PDL1 exhibits excellent synergistic effects in antitumor therapy, which may be attributed to the potent immune activation induced by the PIT/ICB combination treatment. Additional staining was applied to tumor slices for pathological and immunofluorescence examination. TUNEL assay results indicated that the highest tumor cell apoptosis rate was observed after the combination treatment, suggesting higher antitumor efficiency (Figure 7h; Figure S32, Supporting Information). Hematoxylin and eosin (H&E) staining of the tumors further demonstrated that the combination therapy group had the most severe fibrosis and marked cell shrinkage (Figure 7h; Figure S33, Supporting Information). Furthermore, immunohistochemical analysis was used to evaluate the expression of PD‐L1 in tumor tissues in vivo, showing a significant downregulation of PD‐L1 expression in the combination therapy group, indicating its excellent in vivo gene silencing effect (Figure 7i; Figure S34, Supporting Information).

Finally, the biosafety of NS (NS‐Ce6, NS‐PDL1, NS‐Ce6‐PDL1) was evaluated by collecting major organs (heart, liver, spleen, lung, kidney) from different treatment groups for H&E staining. The results showed no significant necrosis in any major organs from all treatment groups, with intact tissue morphology and no lesions, indicating good biosafety of NS (Figure S35, Supporting Information). Moreover, we assessed the biocompatibility of the activatable designed NS through hemolysis experiments, using always‐on NS (NS‐on) as a control to compare their side effects. Compared to NS‐on, the hemolysis rate of NS under laser or sunlight irradiation was negligible (<5%) (Figure S36, Supporting Information). Furthermore, we investigated the in vivo phototoxicity of NS. The results showed that compared to NS‐on, the activatable design of NS could also prevent photodamage to blood cells and vessels during circulation (Figure S37, Supporting Information). These results confirmed that the activatable designed NS had excellent biocompatibility and safety.

## 3 Conclusion

This study developed a smart DNA nanosphere composed of multifunctional modules capable of targeted delivery and controlled release of photosensitizers and immune adjuvants, aiming for efficient synergistic PIT and ICB while minimizing side effects on normal tissues. The NS offers several advantages: 1) Enhanced tumor targeting and controlled activation ensure precise delivery and activation of therapeutic agents, maximizing tumor‐killing efficacy while minimizing side effects. 2) The NS demonstrates synergistic anti‐tumor effects by combining PIT with the release of immune checkpoint inhibitors, not only enhancing tumor cell killing but also promoting a robust immune response by downregulating PD‐L1 expression, thereby reducing immune tolerance. 3) The smart DNA nanoplatform is modular, versatile, and multifunctional, allowing the application to other immune‐related proteins by simply substituting the siRNA targeting specific proteins, providing a novel strategy for precision cancer therapy. Additionally, due to their programmability, safety, and biodegradability, DNA nanomaterials are excellent candidates for smart nanomedicine. This study demonstrates the significant potential of DNA nanotechnology in achieving targeted, safe, biodegradable, and precise drug delivery. The smart DNA nanoplatform exhibits good biocompatibility, excellent photodynamic efficiency, a superior immune response, effective tumor suppression, and low toxicity, making it a promising strategy for combined cancer immunotherapy.

## 4 Experimental Section

### Synthesis and Stability Analysis of NS

To synthesize the NS, the basic units were first prepared consisting of Y‐shaped DNA (Y‐DNA) and linker DNA (L‐DNA). To prepare the Y‐DNA, three partially complementary single‐stranded DNA (Y1, Y2, and Y3) were mixed in equal concentrations. The buffer solution used was PBS containing 50 mm Mg^2^⁺. The solution was heated to 95 °C for 5 min and then cooled to room temperature for 2 h. The same technique was used to prepare L‐DNA, with a concentration ratio of 1:1:2 for L1, L2, and L3. A stock solution of mixed Y‐DNA and L‐DNA (molar ratio of 1:2) was incubated at 37 °C for 24 h. The synthesis of NS was characterized using 2% agarose gel electrophoresis. Transmission electron microscopy and scanning electron microscopy were used to study the size and morphology of NS. For stability analysis, the NS was added to PBS and 10% FBS and incubated at 37 °C for 0, 0.5, 1, 2, 3, and 4 days. The stability was verified using 2% agarose gel electrophoresis.

### Disassembly Analysis of NS

The NS stock solution (10 µm) was diluted to 100 nm in PBS at pH 5.0. Particle size was measured by DLS in PBS at pH 7.4 and 5.0. CD analysis of i‐motif formation, the NS stock solution (100 µm) was diluted to 2.5 µm in PBS at pH 7.4 and pH 5.0. Absorbance was measured at room temperature using a CD chiroptical spectrometer (MOS‐500, France) over a wavelength range of 220–320 nm.

### Acid Response Range of NS

The pH response of NS was evaluated by measuring the fluorescence emission spectra in buffer solutions of different pH. NS stock solution (10 µm) was diluted to 500 nm, and emission spectra were collected from 640 to 740 nm under 620 nm excitation. Fluorescence intensity at 666 nm quantified pH sensitivity efficiency, and fluorescence release kinetics at pH 5.0 were monitored over time.

### RNase H‐Mediated siRNA Release

NS‐PDL1 was incubated with varying concentrations of RNase H (0, 6.25, 12.5, 25, 50, 100, and 200 U mL^−1^) for 1 h at 37 °C. Additionally, NS‐PDL1 was incubated with 100 U mL^−1^ RNase H for different durations. The disassembly of NS and release of siRNA were analyzed using 10% PAGE electrophoresis. Gray values of electrophoretic bands were analyzed using Image J.

### Immunofluorescence Analysis

The cells seeded on confocal dishes were washed with PBS and fixed with methanol (PD‐L1 detection) or 4% paraformaldehyde (HMGB1 and CRT detection). For the visualization of cytoplasmic and nuclear proteins, Cells were permeabilized with 0.5% Triton X‐100, blocked with 5% BSA, and incubated with primary antibodies against PD‐L1, HMGB1, and CRT. After incubation with Alexa Fluor 594‐labeled secondary antibody and Hoechst 33342, images were acquired using CLSM.

### Co‐Culture and T‐Cell Killing Assays

Co‐culture experiments followed established protocols.^[^ ^24^ ^]^ Jurkat T cells were activated with PHA (500 ng mL^−1^) for 72 h before co‐culturing with MCF‐7 cells. CTLL‐2 cells were activated with IL‐2 and co‐cultured with 4T1 cells. After co‐culture, T cells were harvested for RNA extraction and RT‐qPCR analysis.

For the T cell killing assay, MCF‐7 and 4T1 cells were treated with PBS, NS, and NS‐PDL1, then co‐incubated with activated Jurkat T cells or CTLL‐2 cells, respectively, for 24 h. Post‐incubation, T cells were removed, and cancer cells were washed with PBS and analyzed using CLSM or flow cytometry for apoptosis.

### In Vivo Immunological Evaluation

The experimental procedures were adopted from existing literature.^[^ ^11a^ ^]^ Upon the 4T1 subcutaneous tumor volume reaching ≈300 mm^3^, mice were randomly assigned to four groups. Each group received different intravenous injections: 1) PBS, 2) NS‐ Ce6, 3) NS‐PDL1, and 4) NS ‐Ce6 ‐PDL1. Mice in groups 2 and 4 were exposed to laser irradiation (660 nm, 100 mW cm^−2^, 5 min), 30 min and 24 h post‐injection, respectively. Three days post‐administration, the mice were euthanized, and their inguinal lymph nodes and tumor tissues were collected for immunological assessment.

### In Vivo Antitumor Experiments

4T1 tumor‐bearing mice were randomly divided into four groups and intravenously injected with PBS, NS‐Ce6, NS‐PDL1, and NS‐Ce6‐PDL1, at a Ce6 dose of 2.0 mg kg^−1^. Mice were irradiated with a 660 nm laser (100 mW cm^−^^2^) at the tumor sites 30 min and 24 h post‐injection, repeating every two days for four cycles. Tumor growth was monitored every other day, and the tumor volume was calculated using the formula (a × b^2^)/2, where “a” and “b” represent the long and short diameters of the tumor, respectively. Subsequently, mice were euthanized, and tumors were excised, weighed, and subjected to H&E staining.

### Ethical Approval Statement

All animal studies were approved by the Hunan Animal Center (Approval No.: SCXK (Xiang) 2018‐0006). The mice were maintained under specific pathogen‐free conditions with unrestricted access to standard food and water, and all procedures were conducted following the “Experimental Animal Regulations.”

### Statistical Analysis

Data are presented as the mean ± standard deviation (SD) for both in vitro and in vivo assays. The sample size for the mouse tumor treatment experiments was four, while the in vitro experiments had a sample size of 3. Statistical analyses were conducted using SPSS 26.0, employing independent samples t‐tests or one‐way/two‐way ANOVA based on the number and distribution of treatment groups. Statistical significance was set at P < 0.05.

## Conflict of Interest

The authors declare no conflict of interest.

## Supporting information



Supporting Information

## Data Availability

The data that support the findings of this study are available in the supplementary material of this article.
