# Supplementary material for: Sequentially Activated Smart DNA Nanospheres for Photoimmunotherapy and Immune Checkpoint Blockade
Source: Adv Sci (Weinh). 2024 Nov 26;12(3):2410632. doi: 10.1002/advs.202410632 (PMC11744728; doi:10.1002/advs.202410632)
Supplement: Supplementary file 1 — Supporting Information [file ADVS-12-2410632-s001.pdf]

ADVANCED SCIENCE Open Access

Supporting Information

for *Adv. Sci.*, DOI 10.1002/advs.202410632

Sequentially Activated Smart DNA Nanospheres for Photoimmunotherapy and Immune Checkpoint Blockade

*Yu Chen, Yu Guo, Wen Cheng, Jiahao Fan, Jiacheng Li, Jiajia Song, Xiaohai Yang, Kemin Wang and Jin Huang**

Supplementary Information

Sequentially Activated Smart DNA Nanospheres for Photoimmunotherapy and Immune Checkpoint Blockade

Yu Chen, Yu Guo, Wen Cheng, Jiahao Fan, Jiacheng Li, Jiajia Song, Xiaohai Yang, Kemin Wang and Jin Huang*

State Key Laboratory of Chemo/Biosensing and Chemometrics, College of Chemistry and Chemical Engineering, Key Laboratory for Bio-Nanotechnology and Molecular Engineering of Hunan Province, Hunan University, Changsha 410082, P. R. China.

*E-mail: jinhuang@hnu.edu.cn

This PDF file includes:

Experimental Section
Tables S1 to S2
Figures S1 to S37

Table of Contents

Experimental Section S1
Supplementary Tables S4
Table S1. The sequences of DNA oligonucleotides. S4
Table S2. The sequences of primer. S5
Supplementary Figures S6
Figure S1. The structure of Y and L monomers. S6
Figure S2. Analysis of nanospheres (NS) self-assembly. S7
Figure S3. Optimization of incubation time for NS synthesis. S8
Figure S4. Optimization of Y and L ratios for NS synthesis. S9
Figure S5. Stability of NS in PBS. S10
Figure S6. Stability of NS in 10%FBS. S11
Figure S7. Disassembly of NS monomer. S12
Figure S8. Electron spin resonance (ESR) analysis of NS. S13
Figure S9. Acid response kinetics of NS. S14
Figure S10. RNase H-mediated NS disassembly. S15
Figure S11. CLSM analysis of cellular uptake S16
Figure S12. Flow cytometry analysis of cellular uptake. S17
Figure S13. Cellular uptake of different concentrations of NS. S18
Figure S14. Effect of temperature on cellular uptake. S19
Figure S15. Analysis of endocytosis pathways. S20
Figure S16. Evaluation of PD-L1 siRNA silencing effect. S21
Figure S17. Cytotoxic effects of laser irradiation times. S22
Figure S18. Cellular uptake of NS in MCF-10A cells S23
Figure S19. Cytotoxicity of NS on MCF-10A cells. S24
Figure S20. CLSM analysis of NS uptake by 4T1 cells. S25
Figure S21. Flow cytometry analysis of NS uptake by 4T1 cells. S26
Figure S22. High mobility group box 1 (HMGB1) release of MCF-7 cells. S27
Figure S23. HMGB1 release of 4T1 cells. S28
Figure S24. Assessment of phytohemagglutinin (PHA)-induced Jurkat T cell activation. S29
Figure S25. Assessment of CTLL-2 cell activation. S30
Figure S26. Co-culture assay of Jurkat T cells on MCF-7 cells. S31
Figure S27. Co-culture assay of CTLL-2 cells on 4T1 cells. S32
Figure S28. In vivo imaging S33
Figure S29. Ex vivo imaging of organs and tumors. S34
Figure S30. Fluorescent images of tumor slices. S35
Figure S31. In vivo activated imaging. S36
Figure S32. TUNEL staining of tumors. S37
Figure S33. H&E staining of tumors. S38

Figure S34. ICH staining of PD-L1. .... **S39**
Figure S35. H&E staining of organs. **S41**
Figure S36. Hemolysis analysis. **S42**
Figure S37. Phototoxicity analysis. **S43**

Experimental section

Materials. Oligonucleotides used in this study were purchased from Sangon Biotech Co., Ltd., except for Ce6-Y1, Ce6-Y2, and Ce6-Y3, which were obtained from Takara Bio (Kusatsu, Japan). The sequences of oligonucleotides are listed in Supplementary Tab. 1. Chlorin e6 (Ce6) was sourced from Macklin Inc. MTS assay kits were procured from Promega, and endocytosis inhibitors (amiloride, chlorpromazine, genistein) were purchased from Aladdin. Essential cell culture materials, including DMEM, trypsin, and antibiotics, were sourced from Gibco (NY, USA). Various assay kits and reagents, such as Calcein/PI cell viability/cytotoxicity assay, reactive oxygen species assay kit, BCA protein assay kit, and LysoTracker Red, were acquired from Beyotime Co., Ltd. Primary antibody anti-PD-L1 and anti-tubulin were purchased from Proteintech. All analytical grade reagents were used without further purification, and deionized water from a Milli-Q system was used for all solutions.

Measurement of $^1O_2$ generation. The generation of $^1O_2$ was measured using a singlet oxygen sensor green (SOSG) (Meilunbio) according to the instructions. NS (10 μM) was added to a 5 mM SOSG stock solution to reach a final concentration of 100 nM. Fluorescence intensity was measured after 660 nm laser irradiation (100 mW/cm^2) at various times, with excitation and emission wavelengths of 494 nm and 525 nm, respectively.

The production of $^1O_2$ was assessed via electron spin resonance (ESR). Initially, the NS was diluted in PBS at pH 7.4 and pH 5.0 and incubated at 37°C for 6 hours to achieve full disassembly. The samples were then exposed to a 660 nm laser (100 mW/cm^2) for 0, 1, 3, and 5 minutes. Following irradiation, the mixtures were treated with a $^1O_2$ trapping agent, and the resulting samples were examined using ESR analysis.

Cell culture. MCF-7 cells were cultured in DMEM/high glucose (DMEM-H) supplemented with 10% FBS, 100 U/mL penicillin, 100 mg/mL streptomycin, and 50 mg/mL gentamycin sulfate under 5% CO_2 at 37°C. 4T1 and Jurkat cells were cultured in RPMI-1640 supplemented with 10% FBS, 100 U/mL penicillin, 100 mg/mL streptomycin, and 50 mg/mL gentamycin sulfate under 5% CO_2 at 37°C. CTLL-2 cells were cultured in specialized medium consisting of RPMI-1640, 100 U/mL rmIL-2, 10% FBS, and 1% penicillin/streptomycin.

***In vitro* cell uptake of NS.** Cells were seeded in confocal dishes for 24 hours, then washed with PBS and incubated with Cy5-labeled NS (+apt) and NS (-apt) for varying durations. After incubation, Hoechst 33342 (10 μg/mL) was added at 37°C for 15 minutes, and the cells were observed using confocal laser scanning microscopy (CLSM). For flow cytometry analysis, cells were seeded in 24-well plates, and incubated with Cy5-labeled NS at different times or with different concentrations. The cells were washed with PBS and then digested with trypsin. The cells were then collected by centrifugation at 2000 rpm for 4 min and washed twice with PBS. Finally, the cells were dispersed in 200 μL of PBS and analyzed by flow cytometry.

Endocytosis pathway and lysosomal colocalization analysis of NS. For endocytosis pathway analysis, cells were seeded in confocal dishes for 24 hours, pretreated with different concentrations of endocytosis inhibitors for 30 minutes, washed with PBS, and incubated with Cy5-labeled NS for 6 hours. Hoechst 33342 was added and incubated for another 15 minutes. The cells were then washed and observed using CLSM. Endocytosis inhibitors used were amiloride, chlorpromazine, and genistein.

For lysosomal localization studies, cells were incubated with NS at different time points. Subsequently, the cells were stained with LysoTracker Red and Hoechst 33342 for 30 minutes. After

washing, the cells were observed using CLSM.

Analysis of intracellular $^1O_2$ generation. Intracellular $^1O_2$ levels were measured using a reactive oxygen species assay kit. Cells were incubated with different materials (PBS, NS-Ce6, NS-PDL1, and NS-Ce6-PDL1) for 6 hours, followed by 5 minutes of laser irradiation (660 nm, 100 mW/cm²). Cells were then washed, incubated with diluted DCFH-DA at 37°C for 20 minutes, and the fluorescence of DCF was observed with CLSM.

Cell viability assay. Cells were seeded in a 96-well plate for 24 hours, treated with different materials for 6 hours, and then exposed to 5 minutes of laser irradiation (660 nm, 100 mW/cm²). After 24 hours, cells were incubated with MTS reagent in the dark for 1 hour. Cell viability was quantified using a microplate reader to measure absorbance at 499 nm.

Calcein-AM/PI staining. Live/dead cell staining was performed using Calcein-AM and PI. Adherent cells were incubated with different materials for 6 hours, exposed to 5 minutes of laser irradiation (660 nm, 100 mW/cm²), and after 24 hours, stained with Calcein-AM and PI for 30 minutes. Live cells were stained green dead cells were stained red, and observations were made using CLSM.

Cell apoptosis analysis. Cell apoptosis was analyzed using YF488-Annexin V /PI double staining. Cells in a 12-well plate were treated with different materials for 6 hours, replaced with fresh medium, and exposed to 5 minutes of laser irradiation (660 nm, 100 mW/cm²). After 24 hours, cells were collected, washed with PBS, and resuspended in 200 μL of binding buffer. Staining reagents were added and incubated for 30 minutes at room temperature, followed by flow cytometry analysis.

RT-qPCR and Western blot analysis. For RT-qPCR, cells were co-incubated with PBS, NS-Ce6, NS-PDL1, and NS-Ce6-PDL1. Total RNA was extracted using RNAiso Plus reagent and reverse transcribed with PrimeScript™ RT reagent mix (Takara). The cDNA was amplified using TB Green® Premix Ex Taq™ (Takara), with primer sequences detailed in Table S2. The relative mRNA levels were normalized to GAPDH expression using the $2^{-\Delta\Delta ct}$ method.

For Western blot analysis, cells treated with different materials were lysed in cold protein lysis buffer with 1% phenylmethylsulfonyl fluoride (PMSF). After centrifugation at 4 °C, 12,500 rpm for 15 minutes, the supernatant containing proteins was obtained and their concentration was determined using a BCA protein assay kit. The supernatant was then diluted with 6× loading buffer and denatured at 99 °C for 5 minutes. Proteins (20 µg) were separated by sodium dodecyl sulfate-polyacrylamide gel electrophoresis (SDS-PAGE) and transferred to a polyvinylidene difluoride (PVDF) membrane (Millipore, Bedford, USA). Subsequently, the membrane was blocked with 5% skim milk, and incubated with primary antibodies (PD-L1 or tubulin). The membrane was incubated with HRP-conjugated secondary antibodies, detected using an ultra-sensitive chemiluminescence system, and imaged with a Multifunctional Molecular Imaging Analysis system (Azure-C600).

Mouse xenograft tumor model. Female BALB/c mice (6-8 weeks) were purchased from Hunan SJA Laboratory Animal Co., Ltd. To establish the tumor model, 4T1 cells (2×10^6 cells / 100 μL) were subcutaneously inoculated into the thigh of mice. *In vivo* imaging experiments began when tumor volumes reached 200-500 mm³. Anti-tumor therapy experiments began when tumor volumes reached 50-100 mm³, with none exceeding 2000 mm³.

***In vivo* fluorescence imaging**. The tumor-targeting efficacy of Cy5-labeled NS was evaluated via tail vein injection in 4T1 tumor-bearing mice. After anesthetizing with 2% pentobarbital sodium, Cy5-labeled NS (Cy5-Y1: 2μM; 100μL) was administered. Dynamic fluorescence distribution was monitored at various time points (0, 2, 4, 6, 12, and 24 hours) using the IVIS Lumina II system.

Mice were euthanized 24 hours post-administration for ex vivo fluorescence observation in tumors and major organs.

***In vivo* immunological evaluation**. To assess dendritic cells (DCs) maturation, DCs were isolated from lymph nodes and stained with anti-CD11c-FITC, anti-CD80-APC, and anti-CD86-PE antibodies. Flow cytometry was utilized to determine the frequency of $CD11c^+/CD80^+/CD86^+$ DCs.

For the analysis of tumor-infiltrating lymphocytes, excised tumor tissues were digested in a DMEM medium containing collagenase IV and hyaluronidase at 37°C for 2 hours. Cell suspensions were obtained with 75-um cell mesh and lymphocytes were isolated using the Mouse Tumor Infiltrating Lymphocyte Isolation Kit (Solarbio) according to protocol. These lymphocyte suspensions were then incubated with anti-CD3-FITC, anti-CD4-PE, and anti-CD8a-APC antibodies for flow cytometry analysis.

To explore the presence of $IFN\text{-}\gamma^+/CD8^+$ T cells, the collected lymphocyte suspensions underwent blocking with anti-CD3-FITC and anti-CD8a-APC antibodies. Subsequently, permeabilization was carried out using Perm/Wash buffer (YESEN) for 30 minutes, followed by staining with anti-IFN-γ-PE antibody.

Histologic examination. After treatment, organs (heart, liver, spleen, lung, and kidney) and tumors were preserved in 4% paraformaldehyde, embedded in paraffin, sectioned, and stained with H&E for microscopic evaluation.

Immunohistochemical (IHC) staining and TUNEL assay. For ICH staining, tumor sections were deparaffinized, blocked with 5% BSA, and incubated with anti-PD-L1 antibody. After washing with PBS, sections were incubated with HRP-conjugated secondary antibody and developed using a DAB (3,3′-diaminobenzidine) HRP color kit.

Apoptotic cells in tumor tissue were analyzed using the One-Step TUNEL Apoptosis Detection Kit (Red Fluorescent) according to the instructions. Tissue was incubated in TdT reaction buffer, followed by staining for nuclei and imaging using CLSM.

Hemolysis analysis. Fresh red blood cells (RBCs) from mice were collected in test tubes containing anticoagulant and centrifuged at 3000 rpm for 10 minutes. The RBCs were then washed four times with PBS to ensure purity. These purified RBCs, diluted in PBS, were incubated with NS or NS-on for 2 hours at 37 °C. PBS (pH 7.4) was used as the negative control, and H_2O was used as the positive control.

After incubation, the samples were exposed to a 660 nm laser (100 mW/cm², 5 minutes) or sunlight (2 hours). Subsequently, the samples were centrifuged, and the absorbance of the supernatant was measured at 570 nm using a microplate reader. The hemolysis rate (%) was calculated using the following formula:

Hemolysis rate (%) = (A0 - A1)/(A1 - A2) × 100%,

where A0 is the absorbance of the supernatant after sample treatment, A1 is the absorbance after PBS incubation, and A2 is the absorbance after H_2O incubation.

Supplementary tables

Table S1. The sequences of DNA oligonucleotides.

Name	Sequence (5'→3')
Y1	ACGCTGTCCTAACCATGACCGCCGAA CTGCAAGTGGT
Y2	TTCGGCGGTCATGTACTAGATCAGGC CTGCAAGTGGT
Y3	GCCTGATCTAGTAGTTAGGACAGCGT CTGCAAGTGGT
L1(RNA)	GAUUCAAGUUAUCAUUUAAUCCUACUGGCAUUUGCUGAACGCAUU
L2(RNA)	GAUUCAAGUUAUCAUUUAAUAAUGCGUUCAGCAAAUGCCAGUAGG
L3	ATTAAATGATAACTTGAATCTTTTTTCCCCCCTCCCCCCTACCACTTGCA GCTCCCCCCTCCCCCC
L2(DNA)	GATTCAAGTTATCATTTAAT CCTACTGGCATTTGCTGAACGCATT
L3(DNA)	GATTCAAGTTATCATTTAAT AATGCGTTCAGCAAATGCCAGTAGG
L3(T)	ATTAAATGATAACTTGAATCTTTTTTCTTTTTTCTTTTTTACCACTTGCAG CTCTTTTTTCTTTTTT
Y1-CY5	ACGCTGTCCTAACCATGACCGCCGAACTGCAAGTGGT-CY5
L3-BHQ2	ATTAAATGATAACTTGAATCTTTTTTCCCCCCTCCCCCCT(BHQ2)ACCAC TTGCAGCTCCCCCCTCCCCCC
L3(T)-BHQ2	ATTAAATGATAACTTGAATCTTTTTTCTTTTTTCTTTTTT(BHQ2)ACCACT TGCAGCTCTTTTTTCTTTTTT
AS1411	ACCACTTGCAGTTTTTTGGTGGTGGTGGTTGTGGTGGTGGTGG

Table S2. The sequences of primer.

Primers	Forward 5′-3	Reverse 5′-3
hPD-L1	TGGCATTTGCTGAACGCATTT	TGCAGCCAGGTCTAATTGTTTT
GAPDH	CGGAGTCAACGGATTTGGTGGTAT	AGCCTTCTCCATGGTGGTGAAGAC
hIL-2	GAATGGAATTAATAATTACAAGAATCCC	TGTTTCAGATCCCTTTAGTTCCAG
mIL-2	GTGCTCCTTGTCAACAGCG	GGGGAGTTTCAGGTTCCTGTA

Supplementary figures

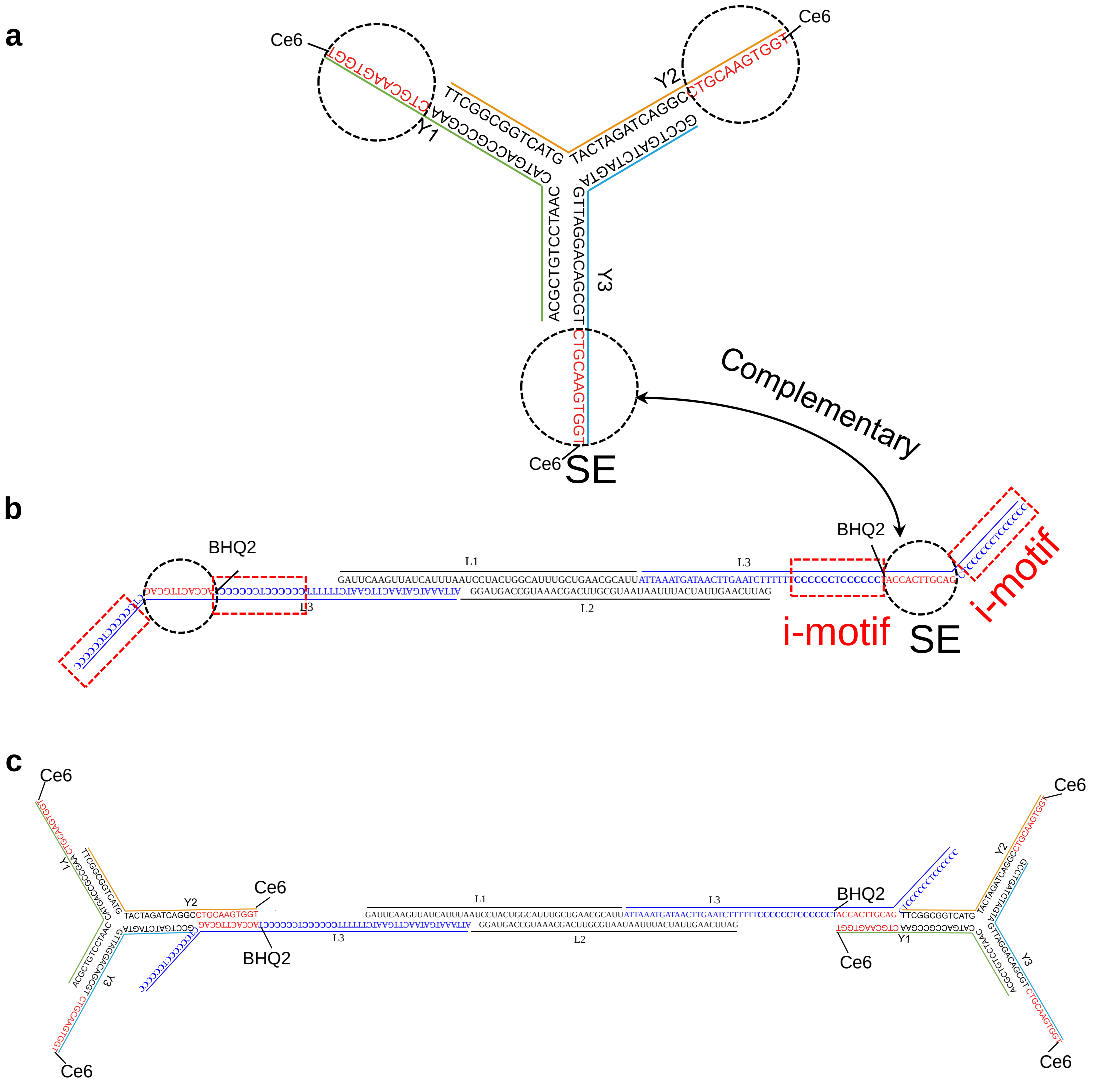

Figure S1. The structure of Y and L monomers.

(a) The structure of Y monomer. **(b)** The structure of L monomer. **(c)** Schematic representation of Y-DNA monomer and L-DNA monomer binding. The black dashed circles highlight the sticky ends (SE) of the Y-DNA and L-DNA monomers, which are complementary. The red dashed box indicates the C-base-rich region that forms the i-motif structure.

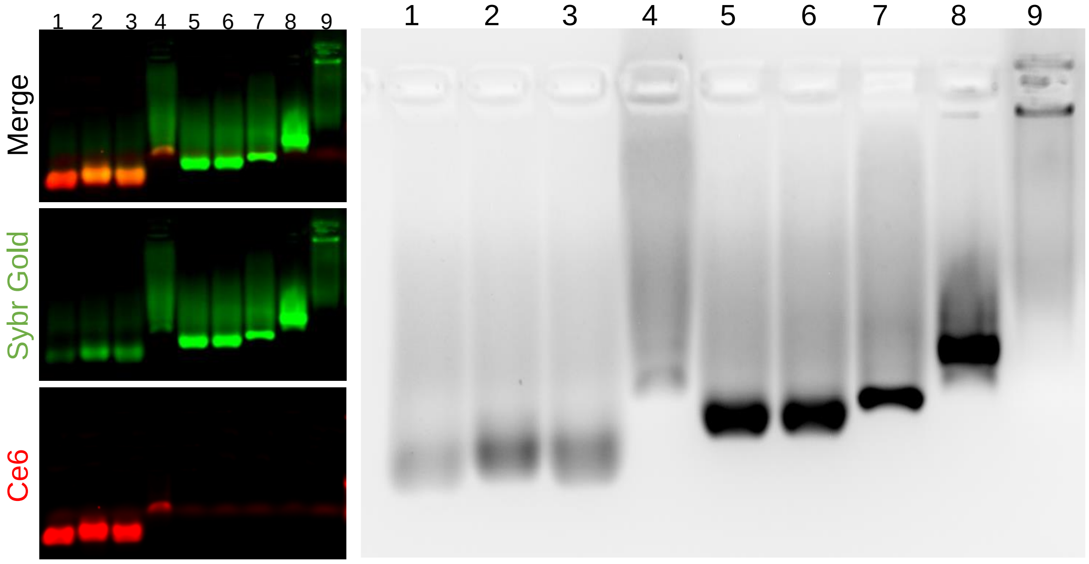

Figure S2. Analysis of NS self-assembly.

2% agarose gel electrophoresis was used to analyze the synthesis of NS. 1:Y1-Ce6, 2:Y2-Ce6, 3:Y3-Ce6, 4:Y-Ce6, 5:L1, 6:L2, 7:L3-BHQ2, 8:L-BHQ2, 9:NS-Ce6-BHQ2. The green fluorescent band represents Sybr Gold-stained unmodified DNA strands, while the red fluorescent band represents DNA modified with Ce6.

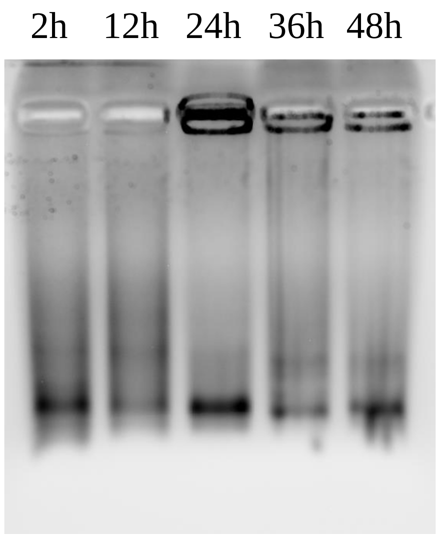

Figure S3. Optimization of incubation time for NS synthesis.

2% agarose gel electrophoresis was used to optimize the incubation time for NS formation. The results showed that NS was successfully synthesized when the incubation time was more than 24 hours.

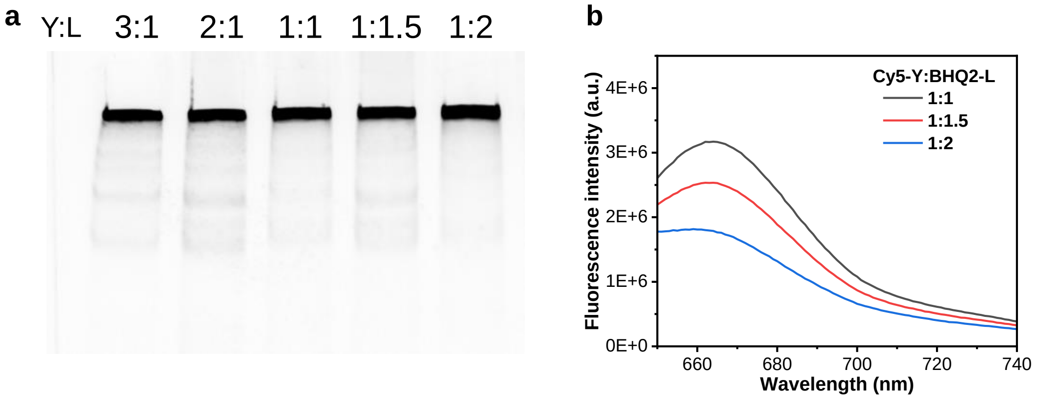

Figure S4. Optimization of Y/L ratios for NS synthesis.

(a)10% PAGE electrophoresis optimized the assembly ratio of Y/L. **(b)** Optimization of the assembly ratio of Y/L by fluorescence method. Electrophoresis results indicated fewer by-products of NS when Y to L was 1:1 and 1:2. Fluorescence results showed a lower fluorescence background for Y/L was 1:2, therefore the concentration ratio of Y/L was set as 1:2 for subsequent experiments.

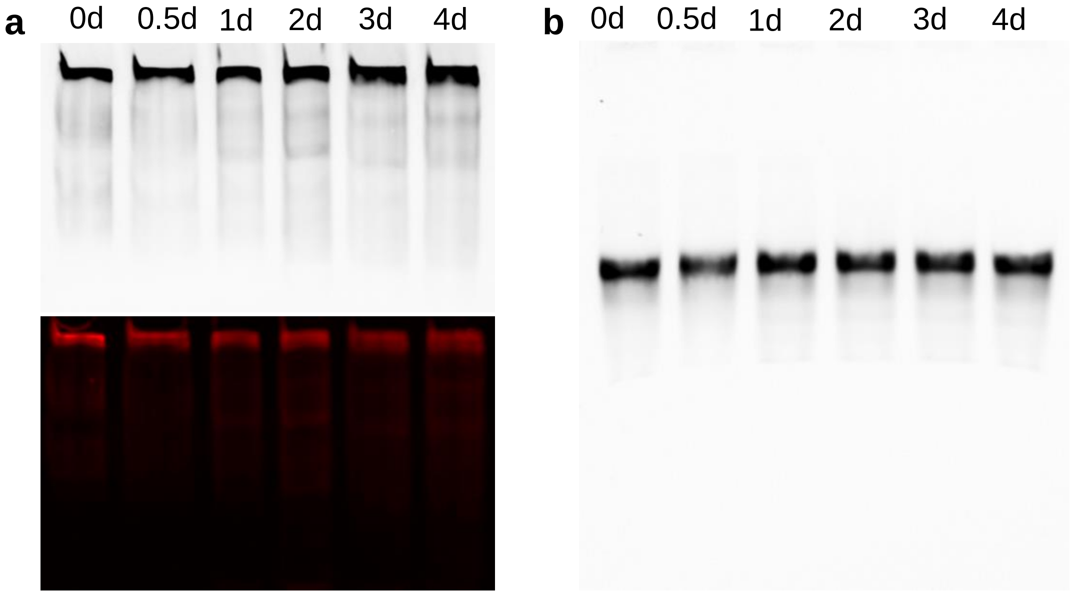

Figure S5. Stability of NS in PBS.

Agarose gel electrophoresis analysis of NS-Ce6**(a)** and L1L2**(b)** after a preassigned incubation in PBS. The red fluorescent band represents DNA modified with Ce6. The results showed NS remained stable in PBS after 4 days, with no significant degradation or change in Ce6 fluorescence. Similarly, no significant changes were observed in L1L2.

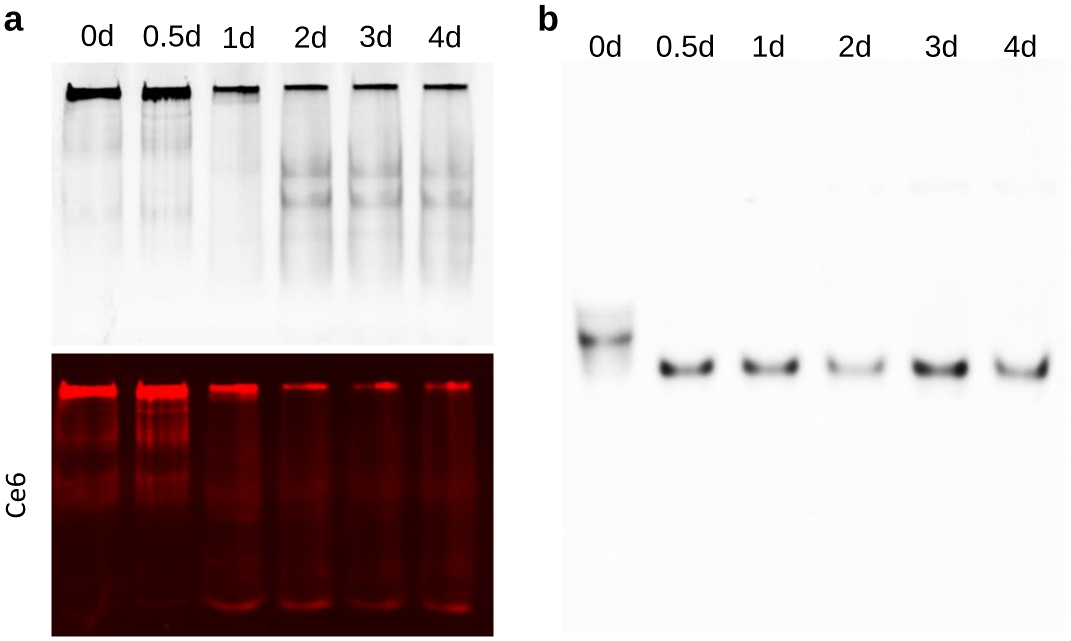

Figure S6. Stability of NS in 10%FBS.

2% agarose gel electrophoresis analysis of NS-Ce6**(a)** and L1L2**(b)** after a preassigned incubation in 10% fetal bovine serum (FBS). The red fluorescent band represents DNA modified with Ce6. The results showed that NS degradation was observed after 4 days in 10% FBS, but NS remained in the loading wells. In contrast, significant degradation of L1L2 occurred after 0.5 days in 10% FBS, suggesting NS provides a protective effect on L1L2.

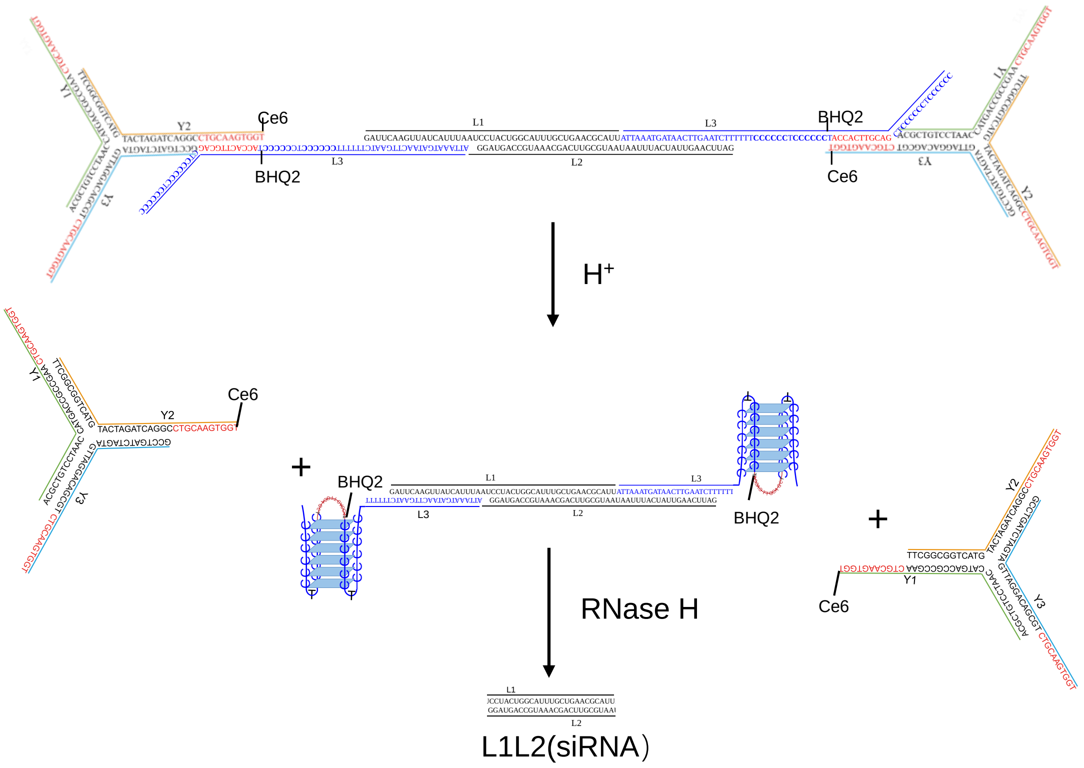

Figure S7. Disassembly of NS monomer.

The acid could induce the transformation of C-rich sequence into i-motif structure, thereby disrupting the hybridization between Y and L, leading to NS disassembly. The L monomer then specifically degrades the RNA sequences in the DNA/RNA hybridized portion in the presence of RNase H, resulting in the release of siRNA (L1L2).

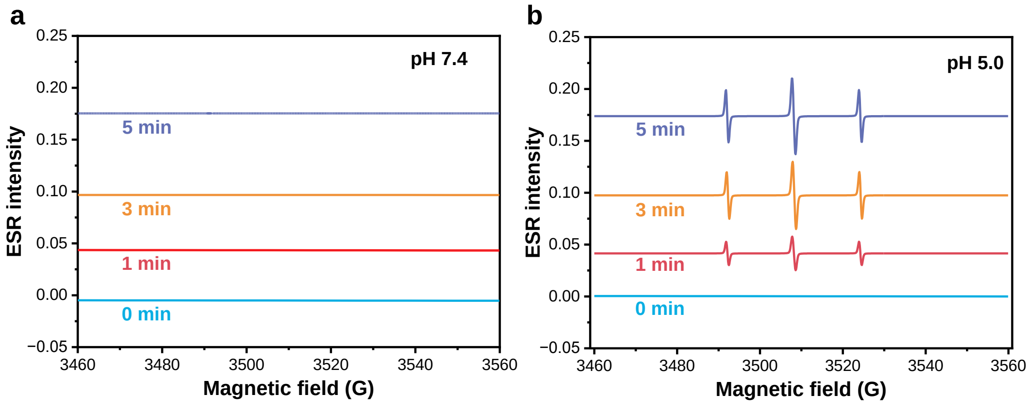

Figure S8. Electron spin resonance analysis of NS.

ESR spectra of NS at pH 7.4 and pH 5.4 during laser irradiation for 0-5 min.

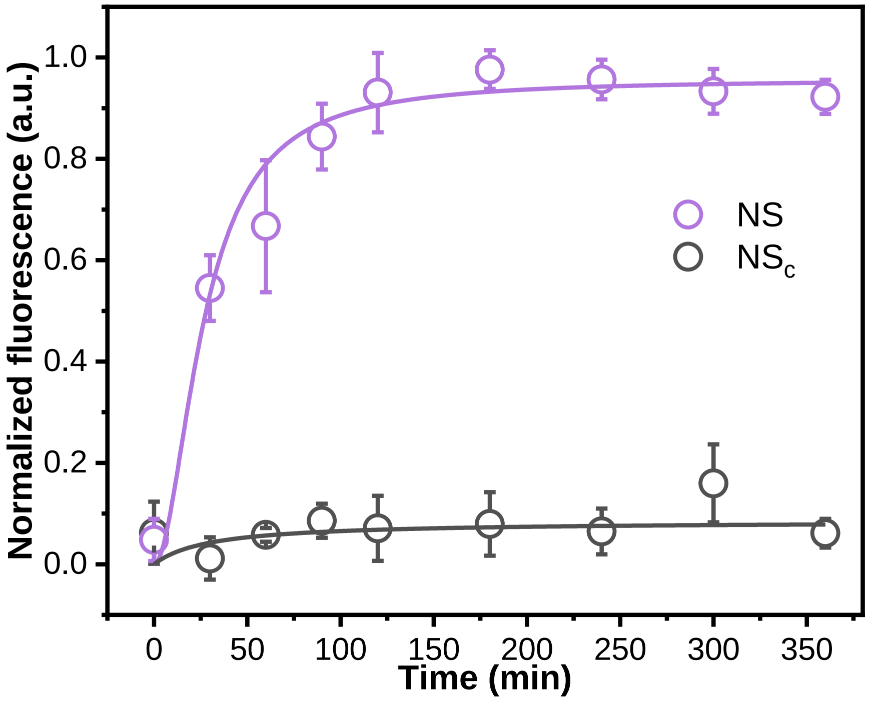

Figure S9. Acid response kinetics of NS.

The acid response kinetics of NS$_c$ and NS at pH 5.0 were examined using a fluorometric assay. The kinetics analysis displayed that the fluorescence of NS rapidly recovered within ~0.5 hours at pH 5.0, whereas NS$_c$ showed minimal fluorescence recovery within 6 hours. Results are presented as means ± SD (n=3).

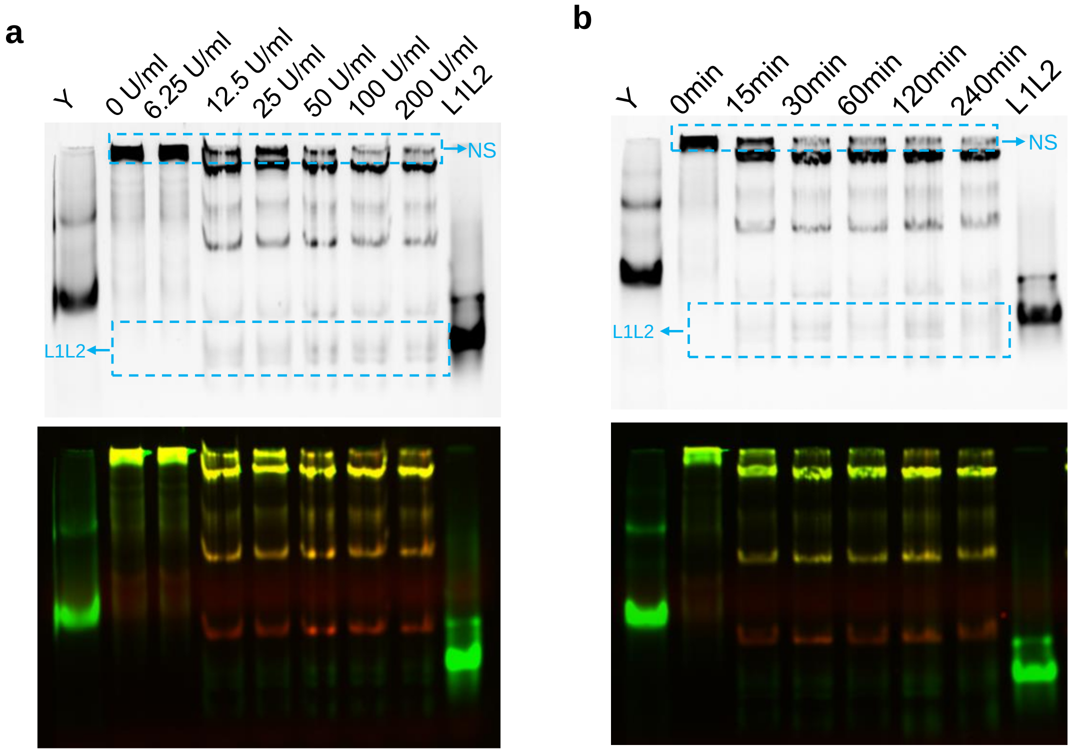

Figure S10. RNase H-mediated NS disassembly.

(a) 10% PAGE analysis of NS-Ce6-PDL1 after incubation with different concentrations of RNase H at 37 °C. **(b)** 10% PAGE gel analysis of NS-Ce6-PDL1 after incubation with 100U/ml RNase H for different times at 37 °C. Green fluorescent bands represent unmodified DNA components and the siRNA linker, which are stained with Sybr Gold. Red fluorescent bands indicate the Ce6-modified DNAs, which are imaged under a 660 nm channel.

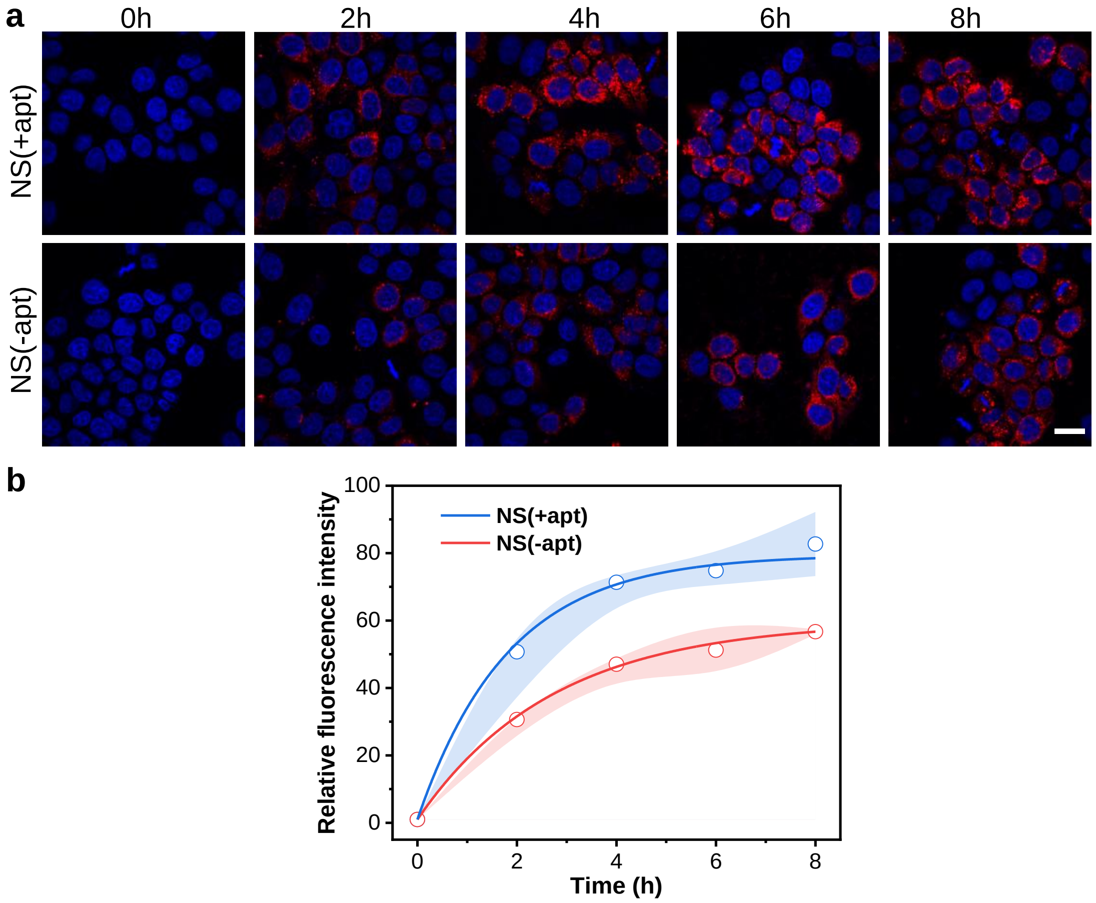

Figure S11. CLSM analysis of cellular uptake.

(a) CLSM images of MCF-7 cells incubated with Cy5-NS(+apt) and Cy5-NS(-apt) at different time points. Scale bar =20 μm. **(b)** The relative fluorescence intensity value of each group in (a). The results showed that the internalization of NS(+apt) -treated cells basically reached saturation at 4 hours, while NS (-apt)-treated cells reached saturation at 6 hours. This indicated that NS(+apt) was taken up by cells more rapidly than NS(-apt). Results are presented as means ± SD (n=3).

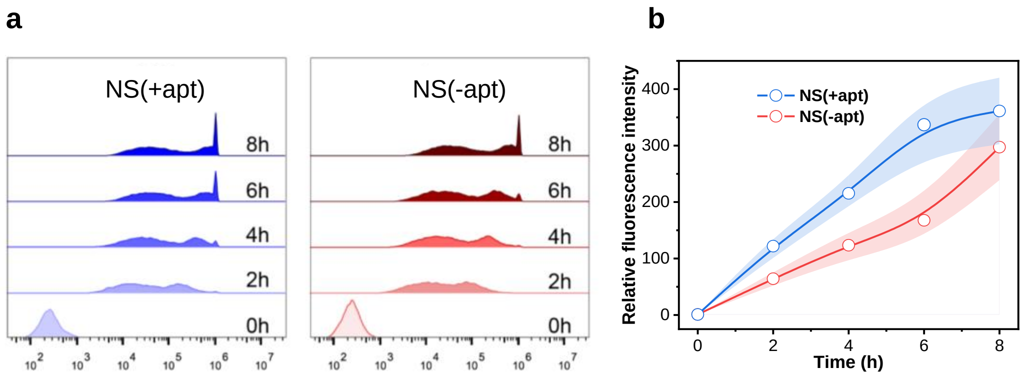

Figure S12. Flow cytometry analysis of cellular uptake.

(a) Flow cytometry analysis of Cy5-NS (+apt) and Cy5-NS (-apt) after incubation with MCF-7 cells for different times. **(b)** Relative fluorescence intensity values for each group in (a). The results showed that NS(+apt) was taken up by cells more rapidly than NS(-apt). Results are presented as means ± SD (n=3).

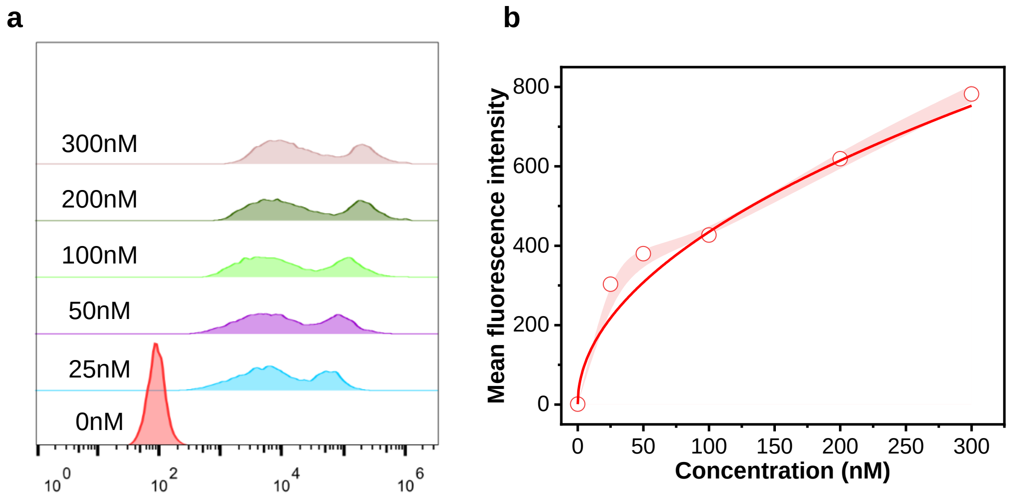

Figure S13. Cellular uptake of different concentrations of NS.

(a) Flow cytometry analysis of MCF-7 cell uptake after incubation with different concentrations of NS. **(b)** Mean fluorescence intensity values of the groups in (a). The results indicated that the uptake of NS was concentration-dependent.

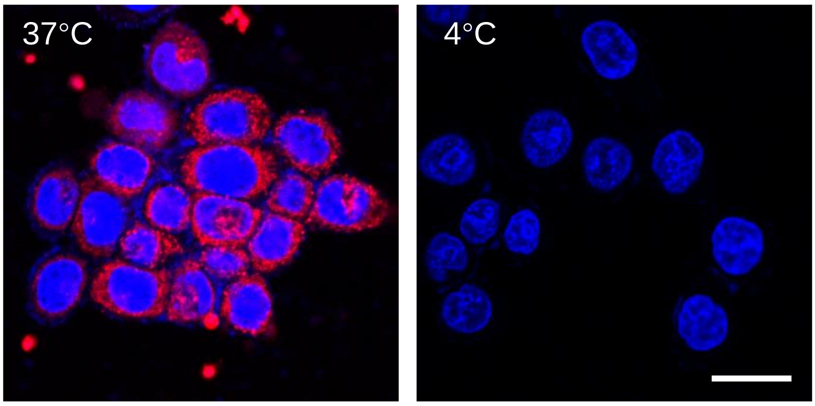

Figure S14. Effect of temperature on cellular uptake.

CLSM analyzed the effect of incubation at 37°C and 4°C on cellular uptake. Scale bar=20 μm. The results showed significant inhibition of cellular uptake at 4°C, indicating that cellular uptake of NS was energy-dependent.

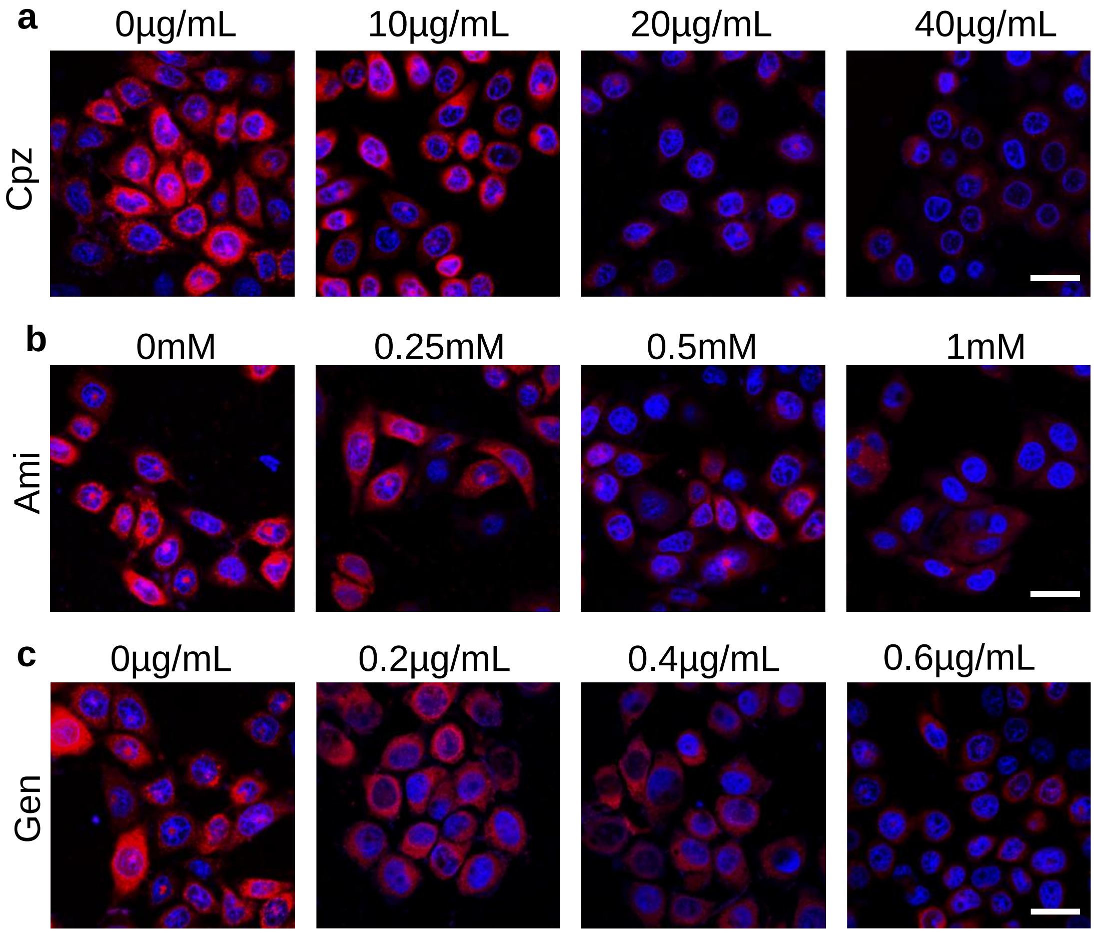

Figure S15. Analysis of endocytosis pathways.

CLSM images of MCF-7 cells treated with three kinds of different concentrations of endocytosis inhibitors and then incubated with NS. **(a)** Chlorpromazine (CPZ). **(b)** Amiloride (Ami). **(c)** Genistein (Gen). Scale bar=20 μm. The results demonstrated that these inhibitors caused a dose-dependent inhibition of endocytosis in MCF-7 cells.

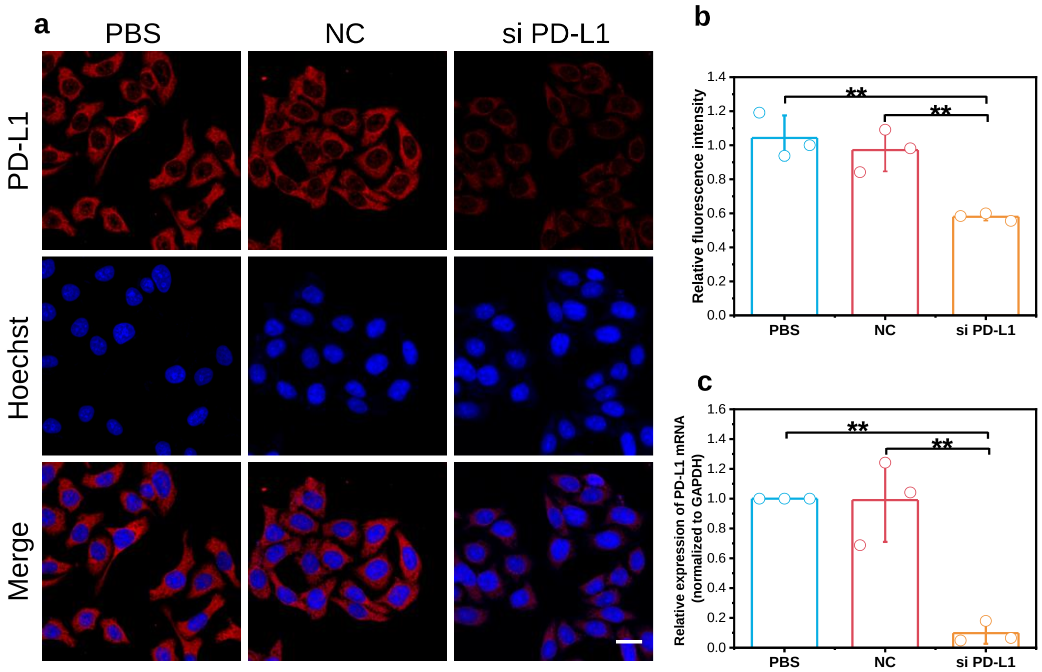

Figure S16. Evaluation of PD-L1 siRNA silencing effect.

Immunofluorescence (IF) and RT-qPCR were employed to demonstrate the gene-silencing effect of siPD-L1 on MCF-7 cells. **(a)** IF detection of the silencing effect of siPD-L1. NC (Negative Control), siPD-L1 (small interfering RNA targeting PD-L1). Scale bar = 20 μm. **(b)** Relative fluorescence intensity statistics corresponding to (a). **(c)** RT-qPCR detection of the gene-silencing effect of siPD-L1. The results indicated that the siPD-L1 group significantly inhibited PD-L1 gene expression in MCF-7 cells compared to the PBS group and NC group. Data are expressed as mean ± SD (n = 3). (**p<0.01; calculated by t-test).

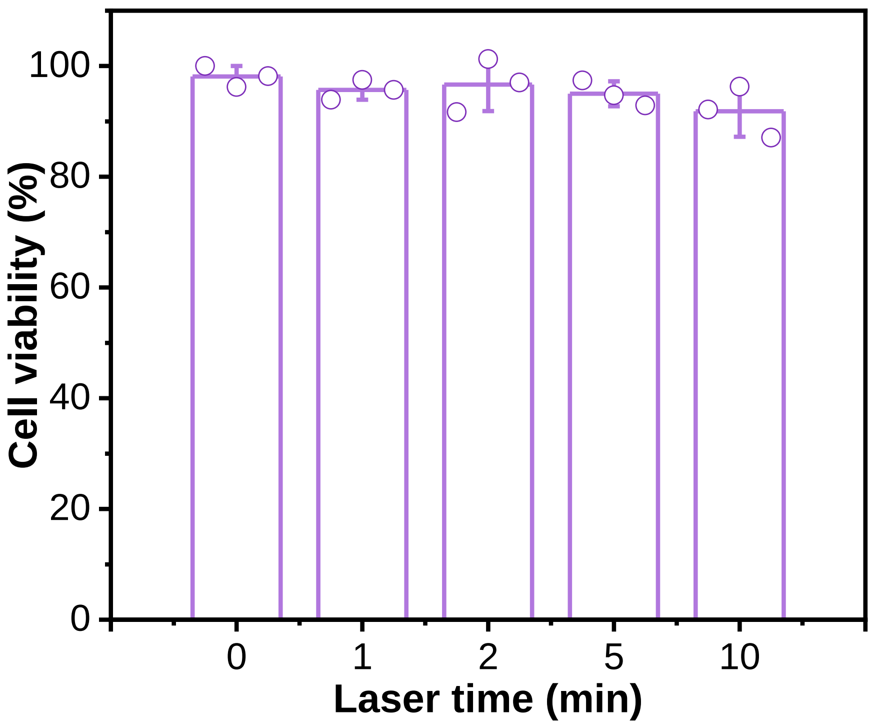

Figure S17. Cytotoxic effects of laser irradiation times.

The cytotoxic effects of different laser irradiation durations on MCF-7 cells were assessed using the MTS assay. The results indicated that exposing MCF-7 cells to 100 mW/cm² laser for up to 10 minutes did not significantly impact cell viability. Results are presented as means ± SD (n=3).

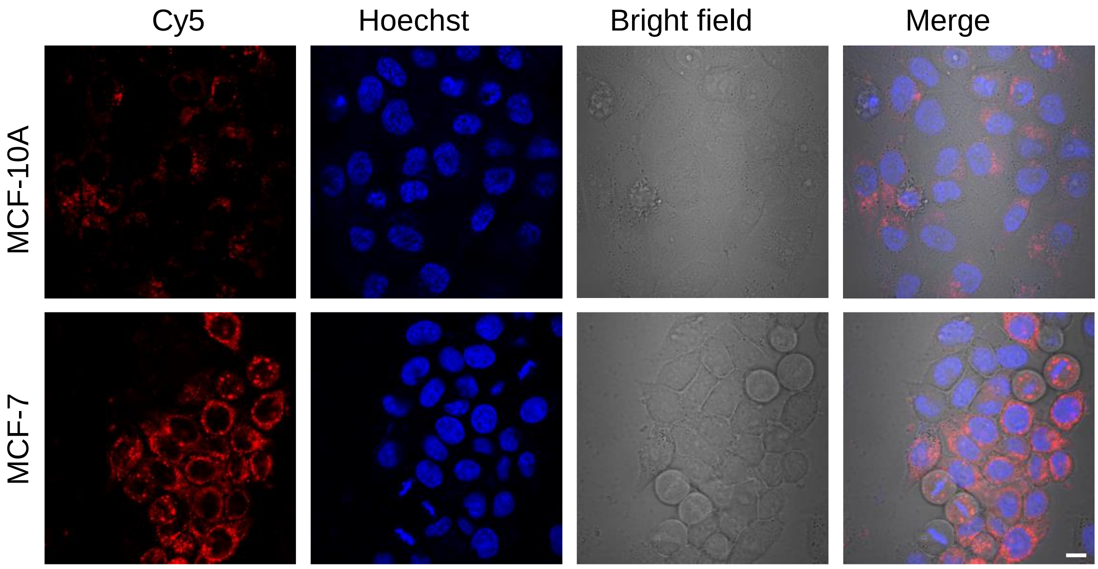

Figure S18. Cellular uptake of NS in MCF-10A cells.

CLSM images of MCF-10A and MCF-7 cells incubated with Cy5-NS for 8 hours. Scale bar = 10 μm. The results showed that the uptake of NS by MCF-10A cells was significantly lower than that by MCF-7 cells.

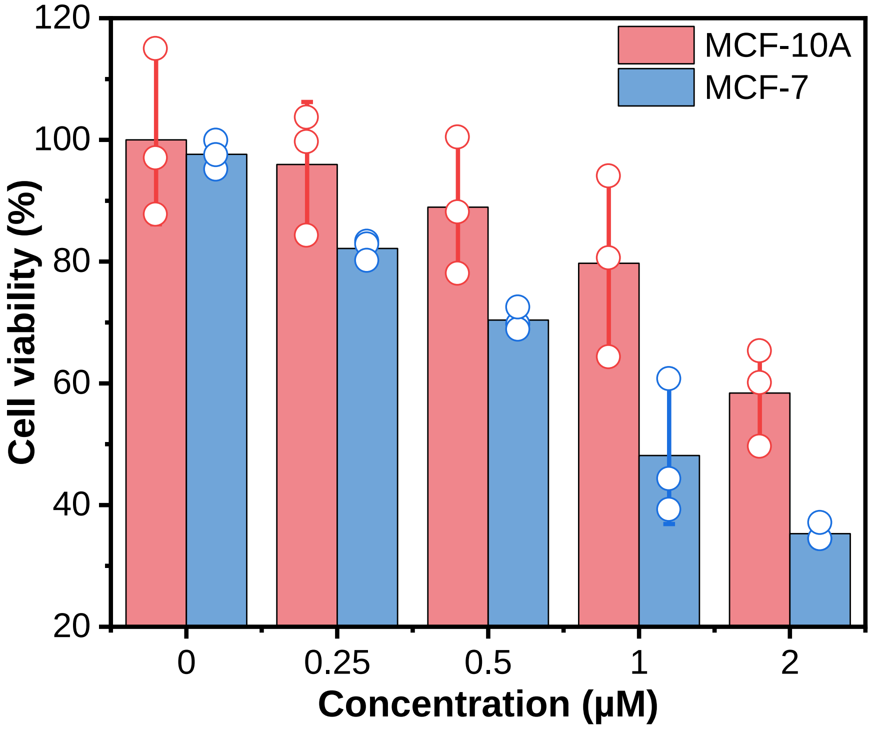

Figure S19. Cytotoxicity of NS on MCF-10A cells.

Cell viability analysis of MCF-10A and MCF-7 cells treated with different concentrations of NS and subjected to laser irradiation. The results showed that NS-Ce6-PDL1 exhibited lower cytotoxicity on MCF-10A cells compared to MCF-7 cells. Results are presented as means ± SD (n=3).

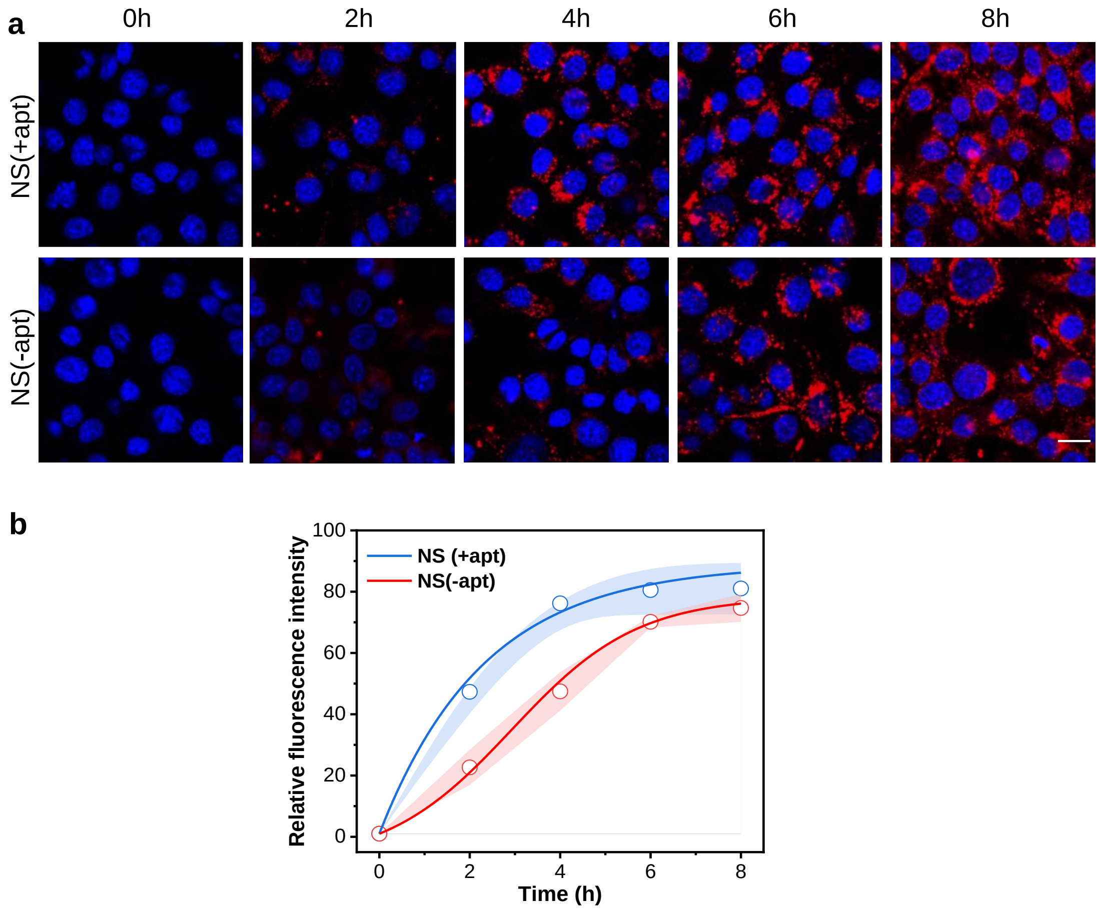

Figure S20. CLSM analysis of NS uptake by 4T1 cells.

(a) CLSM images of 4T1 cells incubated with Cy5-NS (+apt) and Cy5-NS (-apt) at different time. Scale bar =20 μm. **(b)** Quantitative analysis of the relative fluorescence intensity in (a). The results indicated that NS (+apt) was taken up more rapidly by 4T1 cells compared to NS (-apt). Results are presented as means ± SD (n=3).

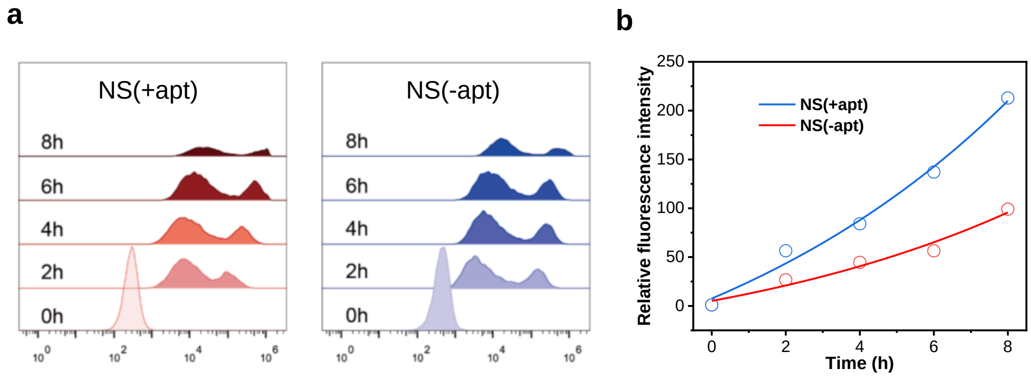

Figure S21. Flow cytometry analysis of NS uptake by 4T1 cells.

(a) Flow cytometry analysis of 4T1 cells uptake after incubation with Cy5-NS (+apt) and Cy5-NS(-apt) for different time. **(b)** Quantitative analysis of the relative fluorescence intensity in (a). Flow cytometry results indicated that NS (+apt) was taken up more rapidly by 4T1 cells compared to NS (-apt).

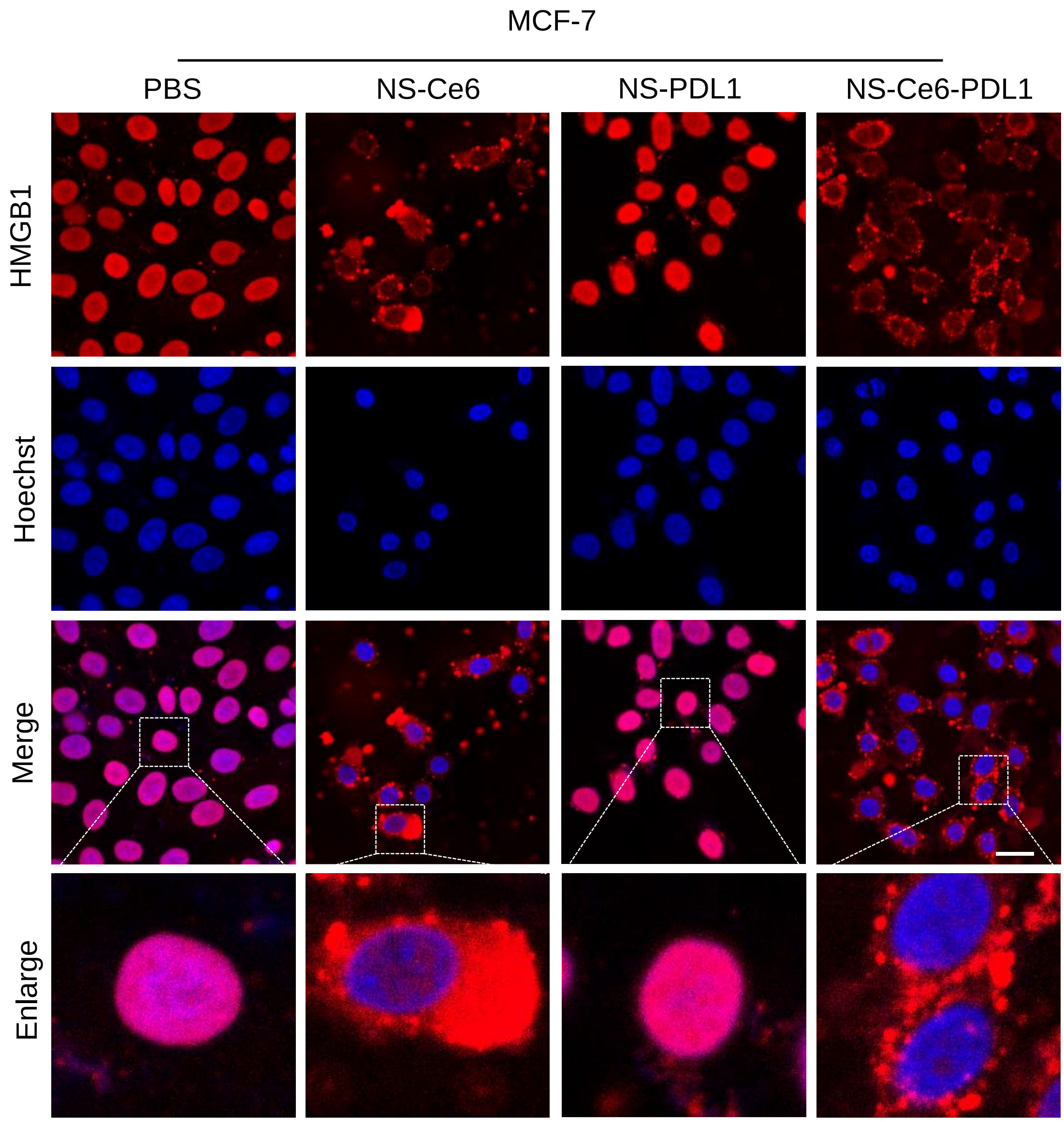

Figure S22. HMGB1 release of MCF-7 cells.

Immunofluorescence staining analysis of HMGB1 release in MCF-7 cells after different treatments. Scale bar=20 μm. The results showed that HMGB1 translocated from the nucleus to the cytoplasm or extracellular environment in MCF-7 cells after NS-Ce6 and NS-Ce6-PDL1 treatments.

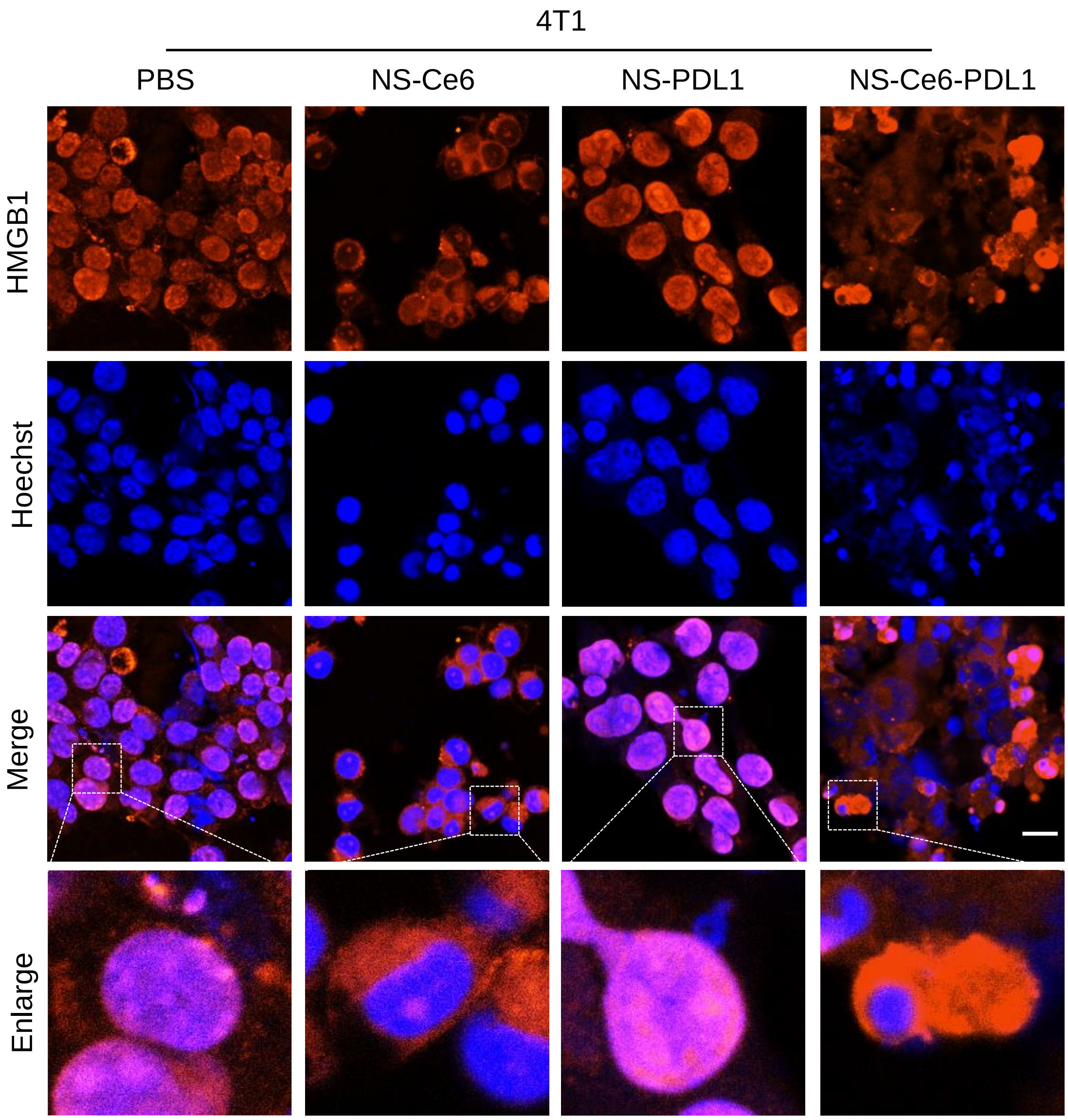

Figure S23. HMGB1 release of 4T1 cells.

Immunofluorescence staining analysis of HMGB1 release in 4T1 cells after different treatments. Scale bar=20 μm. The results showed translocation of HMGB1 from the nucleus to the cytoplasm or extracellular environment in 4T1 cells after NS-Ce6 and NS-Ce6-PDL1 treatments.

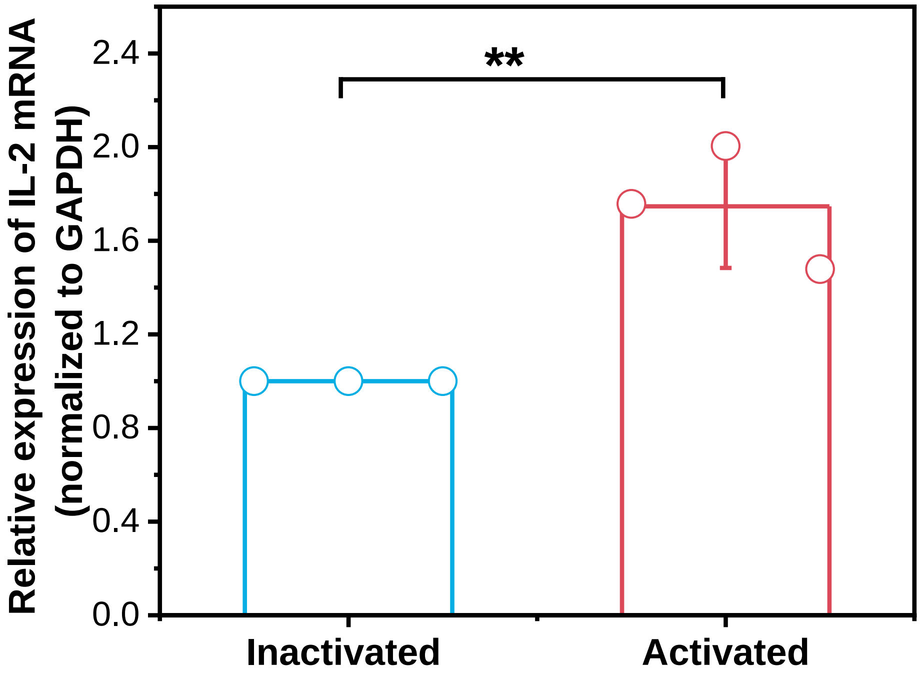

Figure S24. Assessment of PHA-induced Jurkat T cell activation.

The levels of IL-2 mRNA were measured using RT-qPCR to assess PHA-induced activation of Jurkat T cells. Data are expressed as mean ± SD (n = 3) (**$p<0.01$; calculated by t-test). The results showed that the IL-2 mRNA levels in activated Jurkat T cells were significantly higher than in inactivated cells, validating the PHA-induced Jurkat T cells activation.

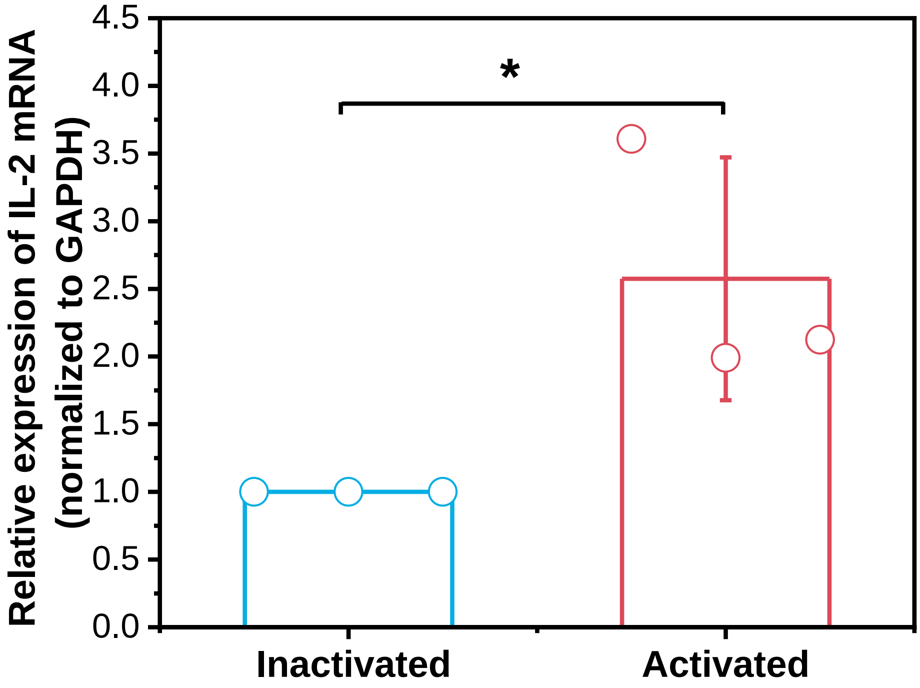

Figure S25. **Assessment of CTLL-2 cell activation.**

The levels of IL-2 mRNA were measured using RT-qPCR to evaluate the activation of CTLL-2 cells. Data are expressed as mean ± SD (n = 3) (*p<0.05; calculated by t-test). The results showed the IL-2 mRNA levels in activated CTLL-2 cells were significantly higher than in inactivated cells, validating the activation of CTLL-2 cells.

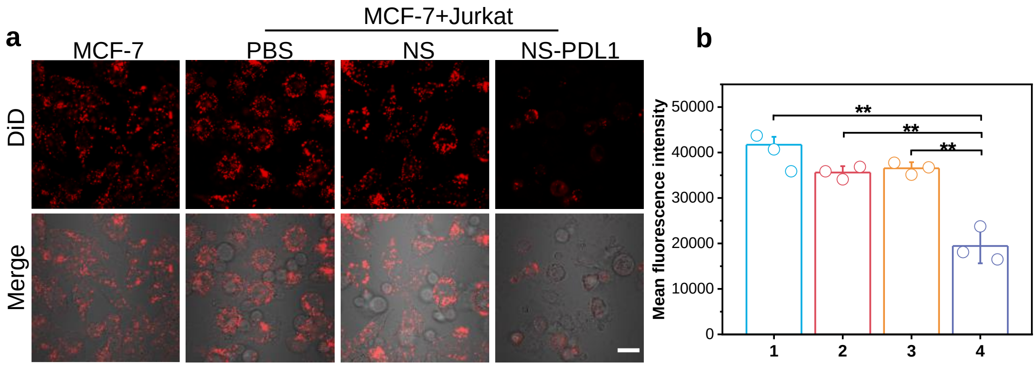

Figure S26. Co-culture assay of Jurkat T cells on MCF-7 cells.

(a) CLSM images verifying the killing effect of Jurkat cells on MCF-7 cells under different treatments. MCF-7 cells were labeled with a Far-red Plasma Membrane Fluorescent Probe (DiD). Scale bar=20 μm. **(b)** Mean fluorescence intensity statistics in (a). Data are expressed as mean ± SD (n = 3) (*p<0.05, **p<0.01; calculated by t-test). Treatments: 1): MCF-7 cells only; 2): Co-culture of PBS-treated MCF-7 with activated Jurkat; 3): Co-culture of NS-treated MCF-7 with activated Jurkat; 4): Co-culture of NS-PDL1-treated MCF-7 with activated Jurkat.

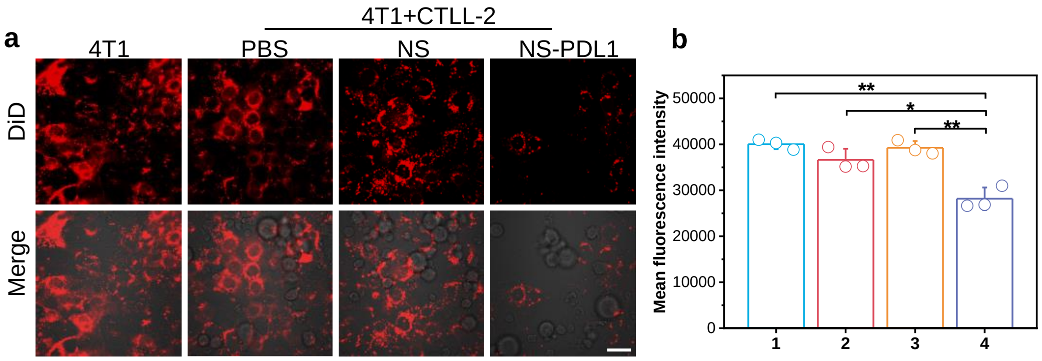

Figure S27. **Co-culture assay of CTLL-2 cells on 4T1 cells.**

(a) CLSM images verifying the killing effect of CTLL-2 cells on 4T1 cells under different treatments. 4T1 cells were labeled with a Far-red Plasma Membrane Fluorescent Probe (DiD). Scale bar=20 μm. **(b)** Mean fluorescence intensity statistics in (a). Data are expressed as mean ± SD (n = 3) (*$p<0.05$, **$p<0.01$; calculated by t-test). Treatments: 1): 4T1 cells only; 2): Co-culture of PBS-treated 4T1 with activated CTLL-2; 3): Co-culture of NS-treated 4T1 with activated CTLL-2; 4): Co-culture of NS-PDL1-treated 4T1 with activated CTLL-2.

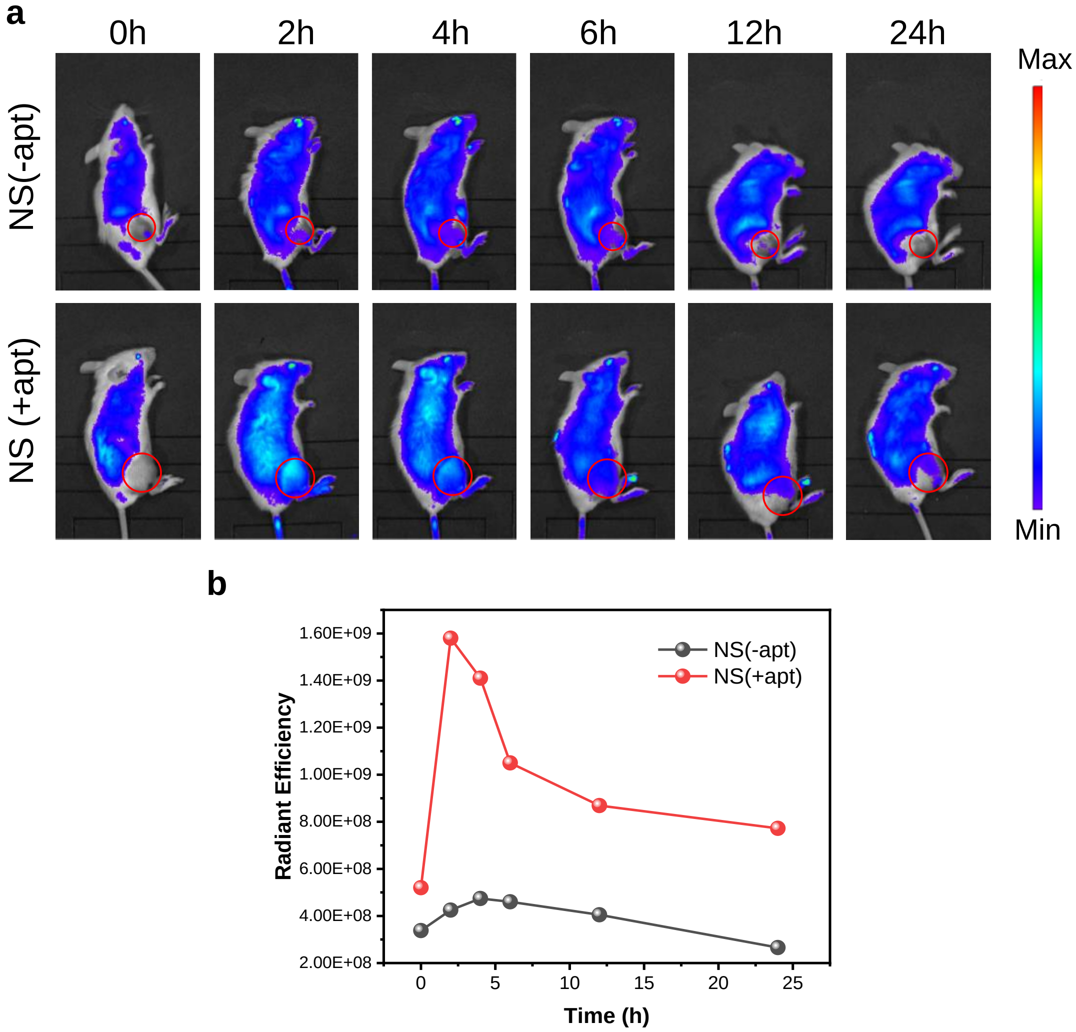

Figure S28. *In vivo* imaging.

(a) *In vivo* biodistribution of Cy5-NS(-apt) and Cy5-NS (+apt) in 4T1 tumor-bearing mice. **(b)** Real-time quantitative analysis of Cy5-NS(-apt) and Cy5-NS (+apt) in 4T1 tumor-bearing mice. The results showed that NS (-apt) had low accumulation efficiency and was rapidly cleared after injection, whereas NS (+apt) reached peak accumulation efficiency at 0.5 hours post-injection and maintained high tumor retention for up to 24 hours.

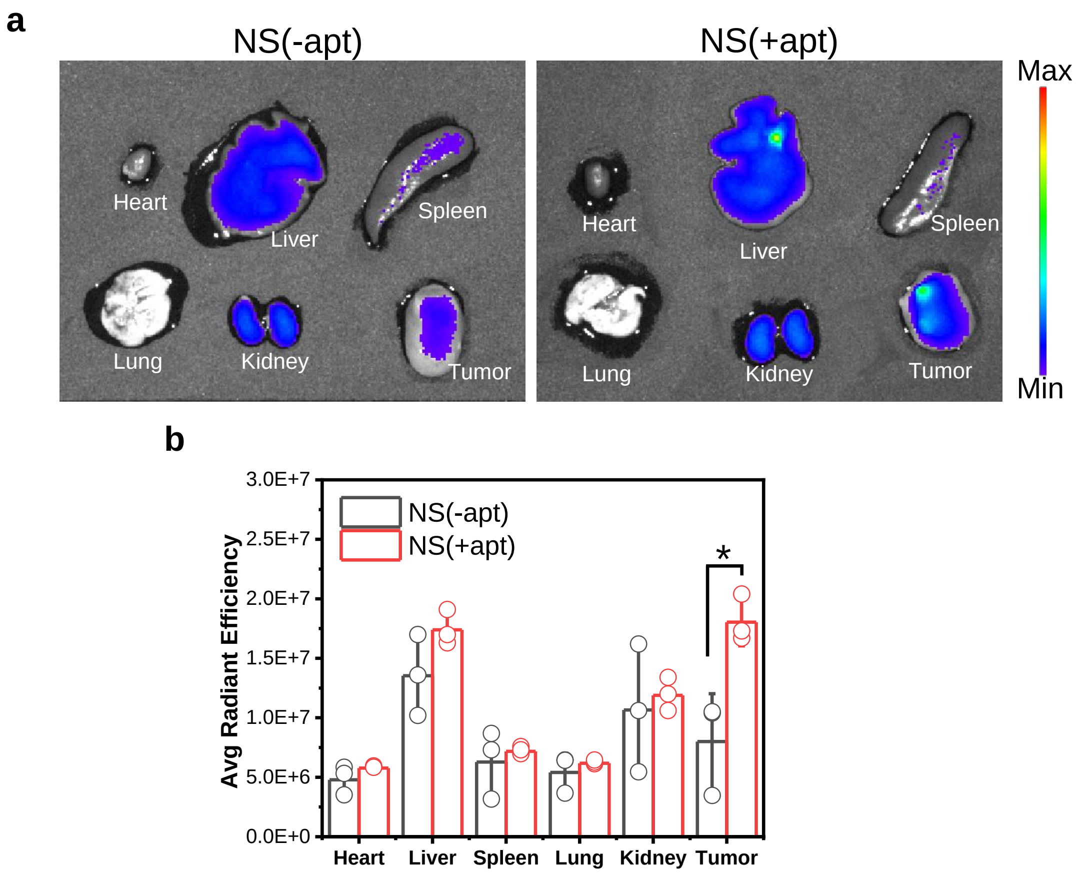

Figure S29. Ex vivo imaging of organs and tumors.

(a) Ex vivo fluorescence images of major organs and tumors after administration of Cy5-NS(-apt) and Cy5-NS (+apt). **(b)** Quantitative analysis of Cy5-NS(-apt) and Cy5-NS (+apt) in major tissues and tumors. Data are presented as mean ± SD (n=3) (*$p<0.05$; calculated by t-test). The results showed that NS could be metabolized by the liver and kidneys, and NS (+apt) significantly accumulated in tumor tissue compared to NS (-apt).

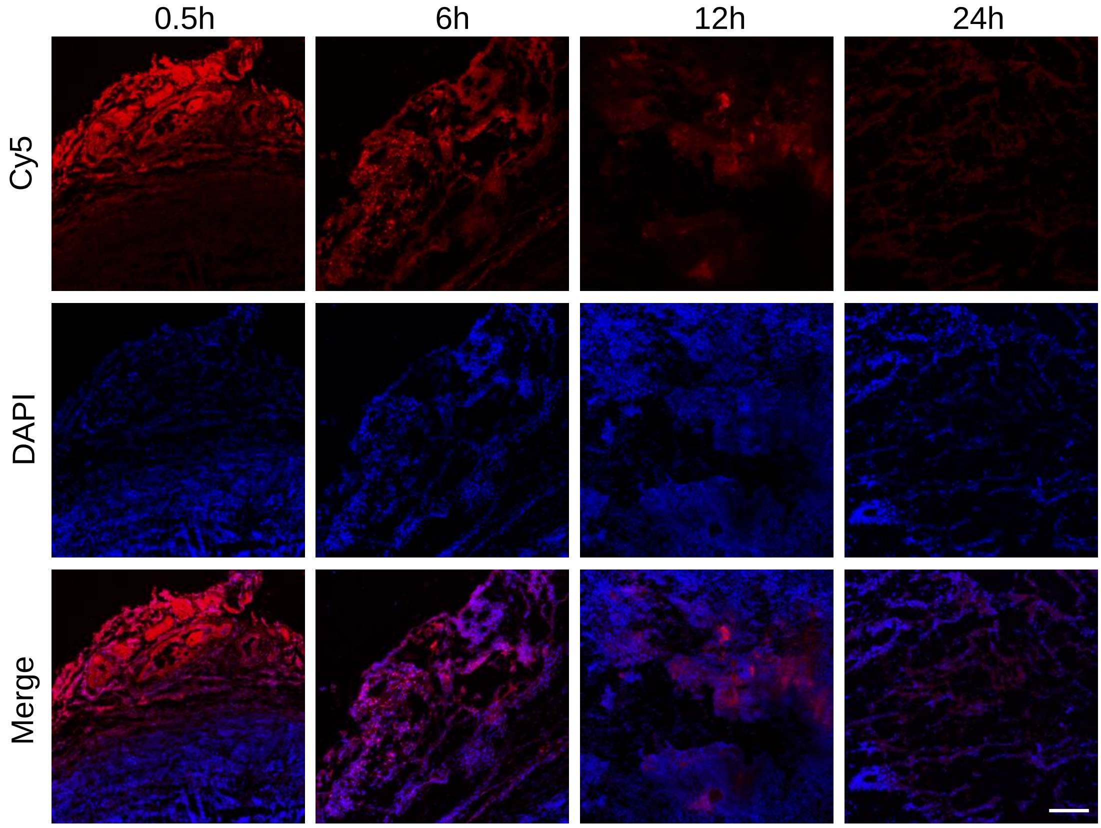

Figure S30. **Fluorescent images of tumor slices.**

CLSM images of tumor slices at different times after intravenous injection of Cy5-NS(+apt) in mice. Scale bar = 200 μm. The results illustrated that NS had a time-dependent distribution at the tumor site and remained fluorescent for up to 24 hours, indicating a relatively long retention time within the tumor.

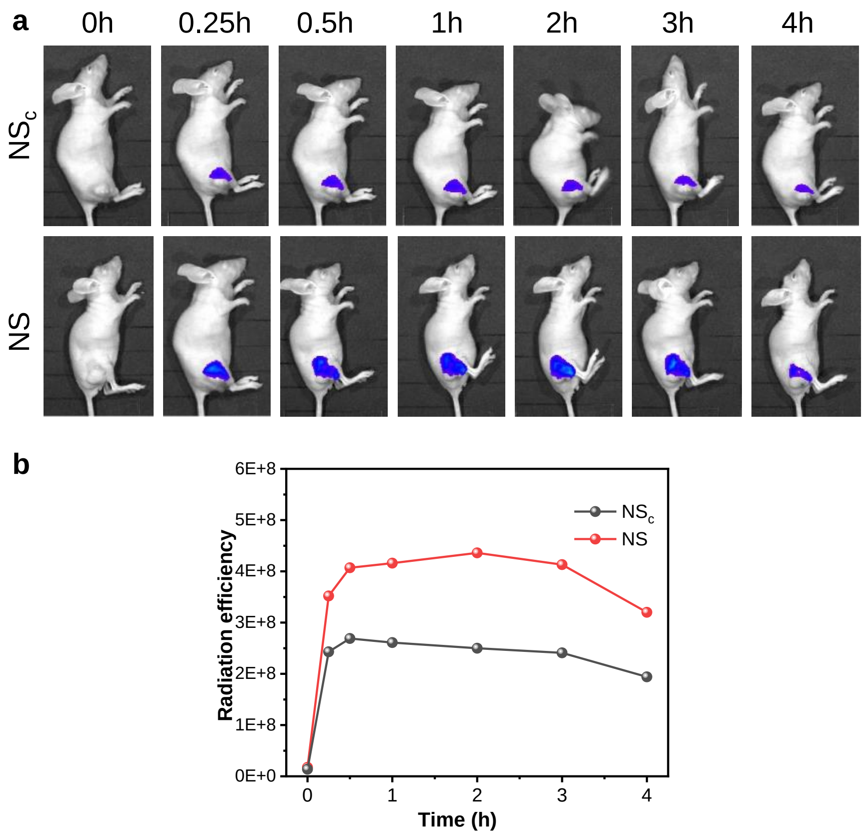

Figure S31. ***In vivo*** **activated imaging.**

(a) In situ activated imaging of NS_c and NS *in vivo* in tumor-bearing mice. **(b)** Quantitative fluorescence analysis of tumor sites.

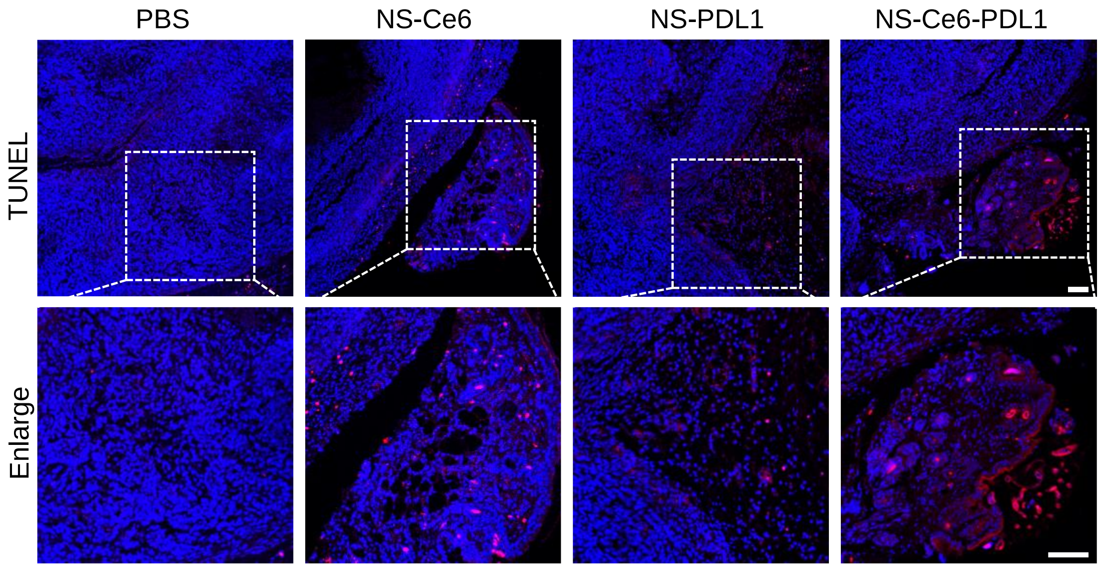

Figure S32. **TUNEL staining of tumors.**

TUNEL staining of tumors at the end of different treatments. Scale bar = 100 μm. The results showed the highest apoptosis rate in tumor cells after combination treatment, indicating higher antitumor efficiency of NS-Ce6-PDL1.

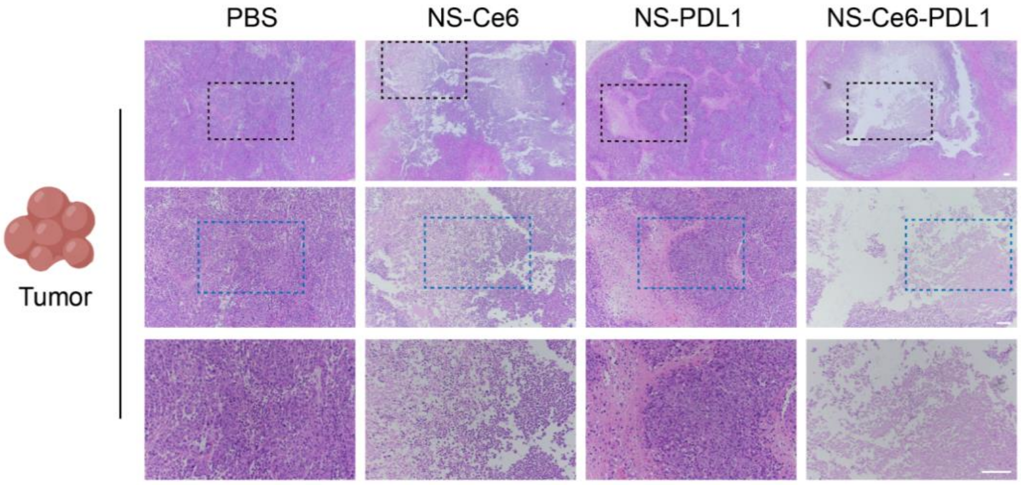

Figure S33. **H&E staining of tumors.**

H&E staining of tumors at the end of different treatments. The images in the second row are enlargements of the black dotted frame sections. The images in the third row are enlargements of the blue dotted frame sections. Scale bar = 100 μm. The results demonstrated that the combination therapy group exhibited the most severe fibrosis and marked cell shrinkage.

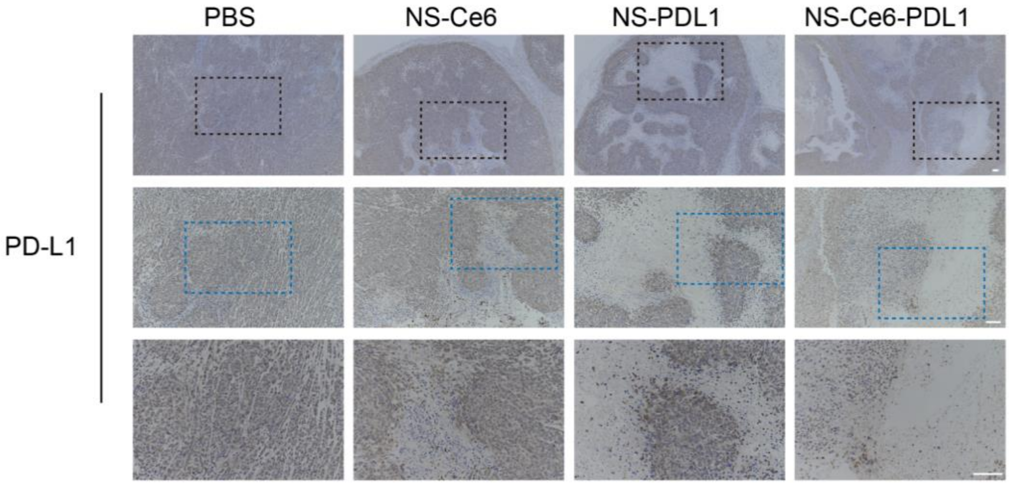

Figure S34. **ICH staining of PD-L1.**

ICH analysis of PD-L1 expression in the tumor at the end of different treatments. The images in the second row are enlargements of the black dotted frame sections. The images in the third row are enlargements of the blue dotted frame sections. Scale bar = 100 μm. The results showed that the expression of PD-L1 in the combination therapy group was significantly down-regulated, suggesting that it has an excellent gene-silencing effect *in vivo*.

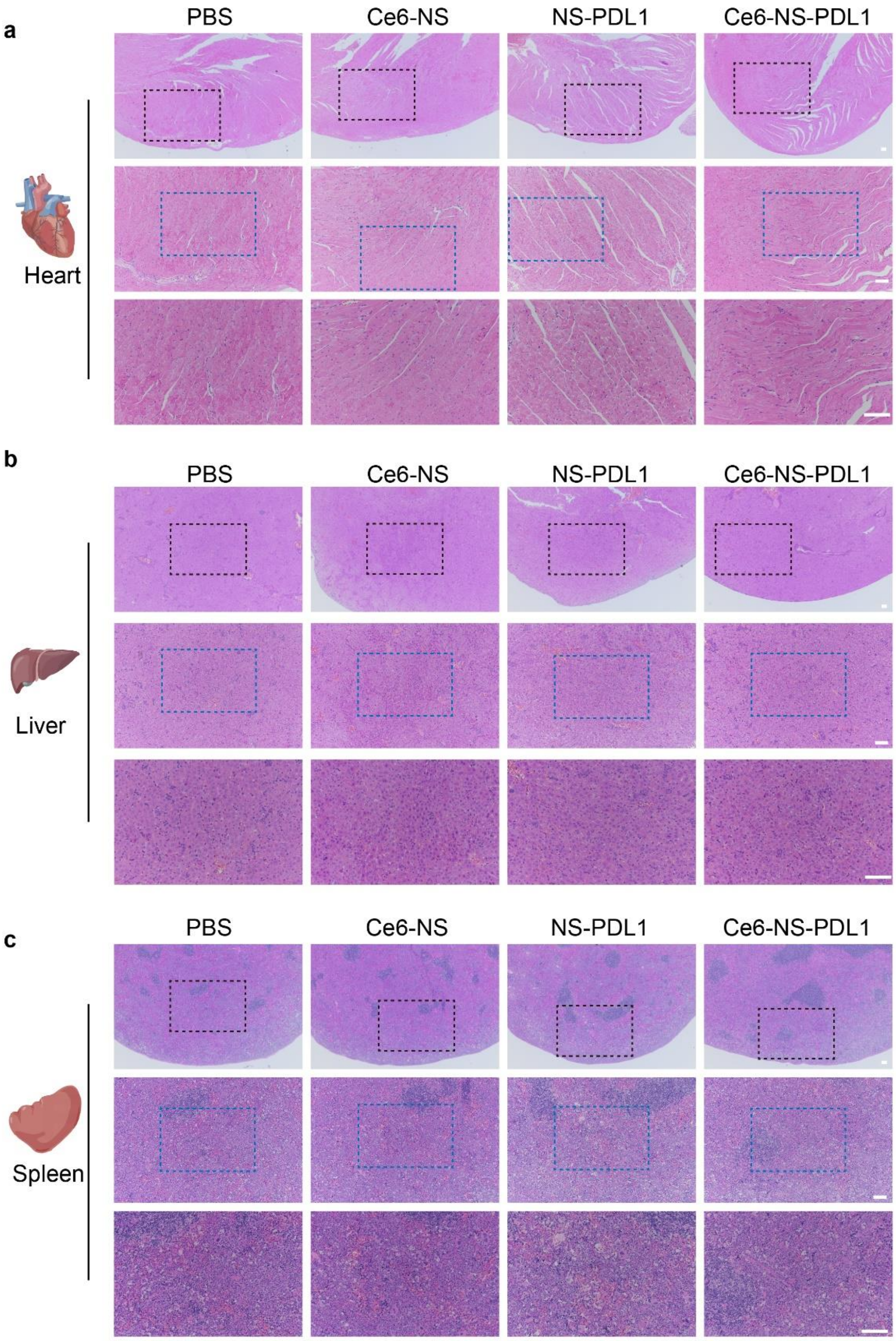
a
PBS
Ce6-NS
NS-PDL1
Ce6-NS-PDL1
Heart
b
PBS
Ce6-NS
NS-PDL1
Ce6-NS-PDL1
Liver
c
PBS
Ce6-NS
NS-PDL1
Ce6-NS-PDL1
Spleen

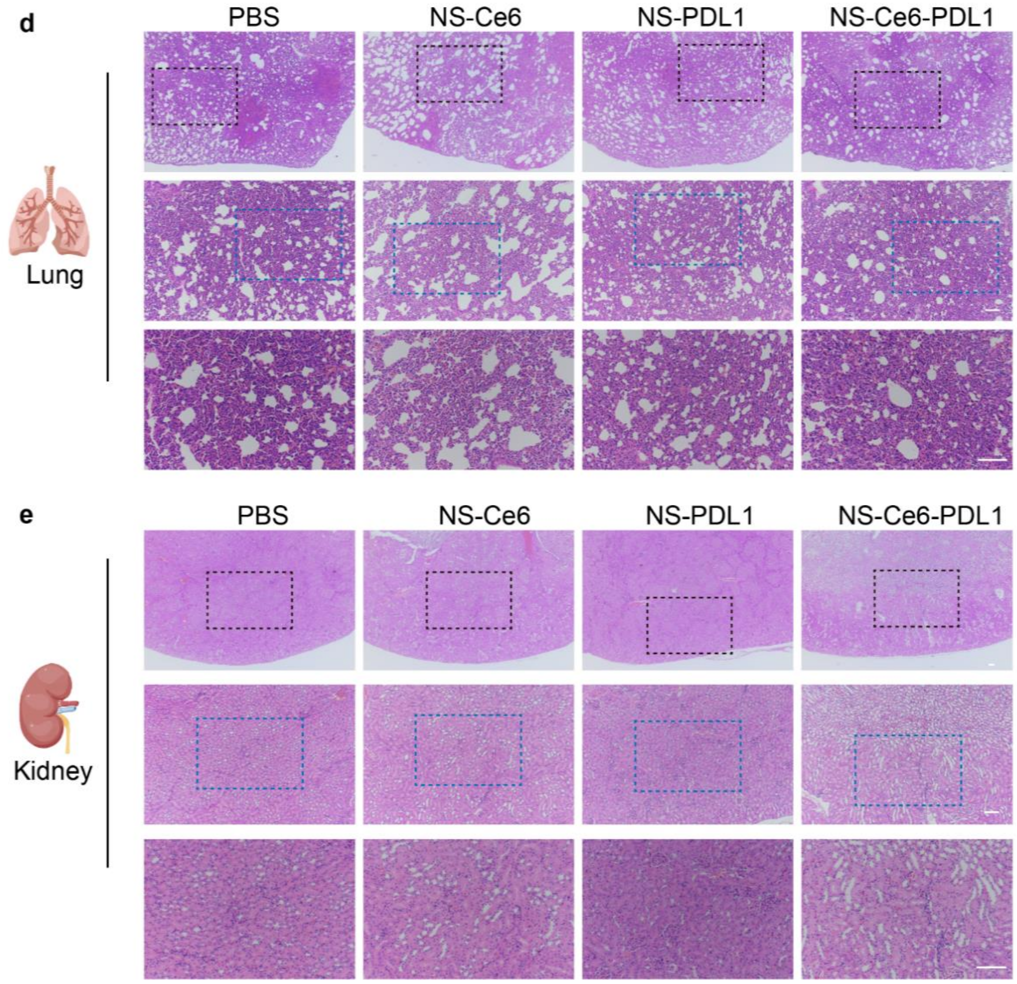

Figure S35. **H&E staining of organs.**

H&E staining of organs at the end of different treatments. **(a)** heart; **(b)** liver;**(c)** spleen; **(d)** lung; **(e)** kidney. The images in the second row are enlargements of the black dotted frame sections. The images in the third row are enlargements of the blue dotted frame sections. Scale bar = 100 μm. The results showed no obvious necrosis in all major organs (heart, liver, spleen, lung, and kidney), with intact and lesion-free tissue slices, verifying the biosafety of NS.

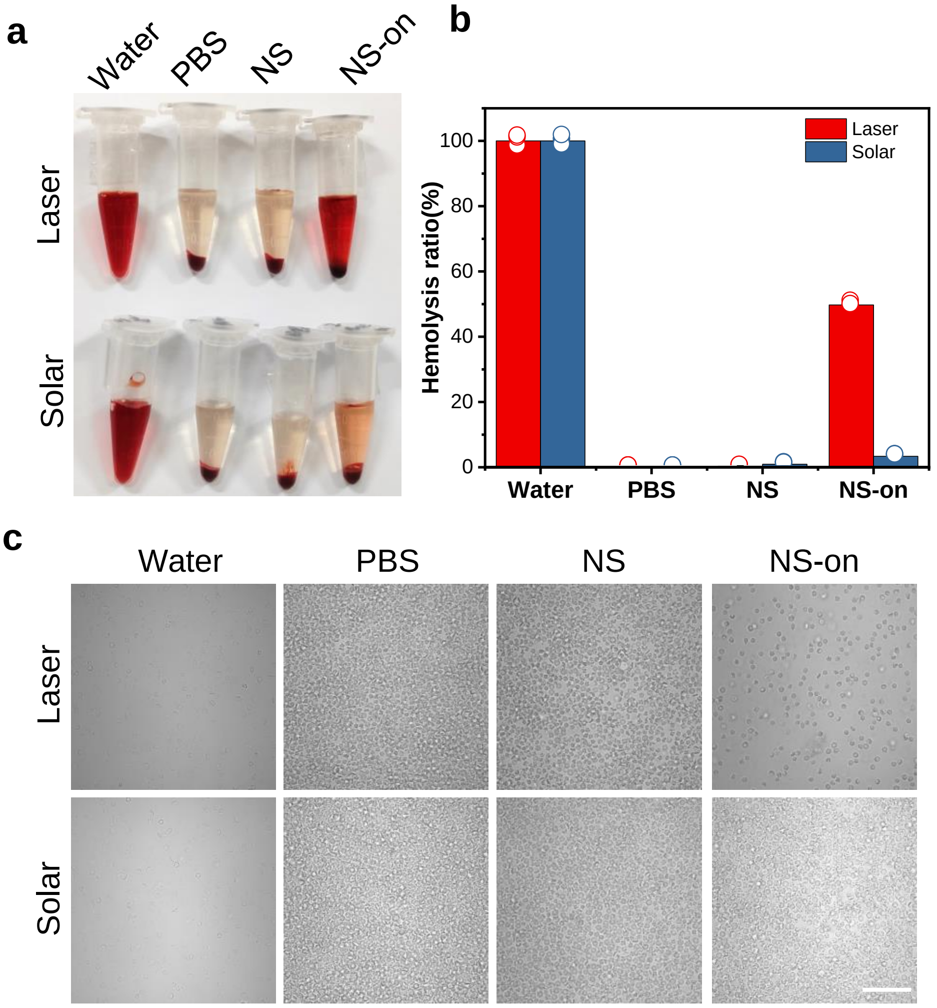

Figure S36. Hemolysis analysis.

(a) Hemolysis analysis of the red blood cells after treatment with NS and NS-on in PBS solution with laser irradiation (100 mW/cm² for 5 min) or solar irradiation (1 h). **(b)** Quantification of the percentage of hemolysis by hemoglobin release into the medium. **(c)** CLSM images of hemolysis analysis of red blood cells after different treatments. Scale bar = 50 μm. (*p<0.05, **p<0.01, ***p<0.001; calculated by t-test). The results showed that NS-on exhibited significant hemolysis under laser irradiation. It suggested that the activatable design of NS offers favorable biosafety compared to NS-on.

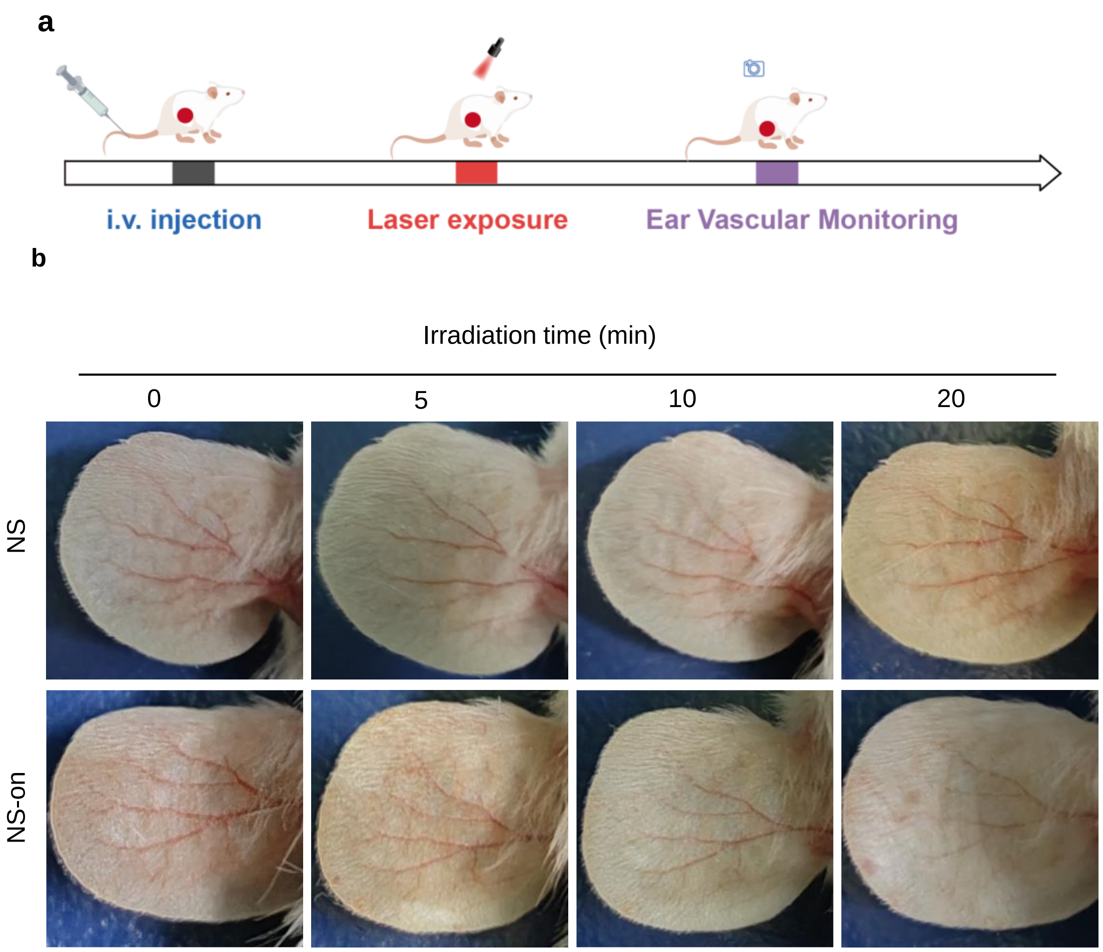

Figure S37. Phototoxicity analysis.

(a) Experimental procedure for *in vivo* phototoxicity assessment. **(b)** Photodamage in ear veins was recorded at indicated times. Mice were injected intravenously with NS and NS-on, then irradiated at 660 nm (100 mW/cm² for 5 min) for varying periods. The results showed severe damage to blood vessels by NS-on. In contrast, the activatable design of NS eliminated photodamage to blood cells and vessels caused by irradiation during circulation.
